# Supplementary material for: Understanding the importance of non-material factors in retaining community health workers in low-income settings: a qualitative case-study in Ethiopia
Source: BMJ Open. 2020 Oct 8;10(10):e037989. doi: 10.1136/bmjopen-2020-037989 (PMC7545659; doi:10.1136/bmjopen-2020-037989)
Supplement: Author's manuscript [file bmjopen-2020-037989.draft_revisions.pdf]

BMJ Open is committed to open peer review. As part of this commitment we make the peer review history of every article we publish publicly available.

When an article is published we post the peer reviewers' comments and the authors' responses online. We also post the versions of the paper that were used during peer review. These are the versions that the peer review comments apply to.

The versions of the paper that follow are the versions that were submitted during the peer review process. They are not the versions of record or the final published versions. They should not be cited or distributed as the published version of this manuscript.

BMJ Open is an open access journal and the full, final, typeset and author-corrected version of record of the manuscript is available on our site with no access controls, subscription charges or pay-per-view fees (http://bmjopen.bmj.com).

If you have any questions on BMJ Open's open peer review process please email info.bmjopen@bmj.com

Understanding the importance of non-material factors in retaining community health workers in low-income settings: A qualitative case-study of Ethiopia

Journal:	*BMJ Open*
Manuscript ID	bmjopen-2020-037989
Article Type:	Original research
Date Submitted by the Author:	29-Feb-2020
Complete List of Authors:	Arora, Nikita; London School of Hygiene & Tropical Medicine, Department of Global Health and Development<br>Hanson, Kara; London School of Hygiene & Tropical Medicine, Department of Global Health Development<br>Spicer, Neil; London School of Hygiene & Tropical Medicine, Department of Global Health and Development<br>Estifanos, Abiy; Addis Ababa University School of Public Health, School of Public Health<br>Keraga, Dorka; Addis Ababa University, School of Public Health<br>Welearegay, Alemtsehay; Mekelle University, School of Public Health<br>Tela, Freweini; Mekelle University, School of Public Health<br>Hussen, Yemisrach ; Addis Ababa University, School of Public Health<br>Mandefro, Yordanos ; Addis Ababa University, School of Public Health<br>Quaife, Matthew; London School of Hygiene and Tropical Medicine Faculty of Infectious and Tropical Diseases, Faculty of Public Health and Policy
Keywords:	Human resource management < HEALTH SERVICES ADMINISTRATION & MANAGEMENT, Health policy < HEALTH SERVICES ADMINISTRATION & MANAGEMENT, QUALITATIVE RESEARCH

SCHOLARONE™
Manuscripts

I, the Submitting Author has the right to grant and does grant on behalf of all authors of the Work (as defined in the below author licence), an exclusive licence and/or a non-exclusive licence for contributions from authors who are: i) UK Crown employees; ii) where BMJ has agreed a CC-BY licence shall apply, and/or iii) in accordance with the terms applicable for US Federal Government officers or employees acting as part of their official duties; on a worldwide, perpetual, irrevocable, royalty-free basis to BMJ Publishing Group Ltd ("BMJ") its licensees and where the relevant Journal is co-owned by BMJ to the co-owners of the Journal, to publish the Work in this journal and any other BMJ products and to exploit all rights, as set out in our licence.

The Submitting Author accepts and understands that any supply made under these terms is made by BMJ to the Submitting Author unless you are acting as an employee on behalf of your employer or a postgraduate student of an affiliated institution which is paying any applicable article publishing charge ("APC") for Open Access articles. Where the Submitting Author wishes to make the Work available on an Open Access basis (and intends to pay the relevant APC), the terms of reuse of such Open Access shall be governed by a Creative Commons licence – details of these licences and which Creative Commons licence will apply to this Work are set out in our licence referred to above.

Other than as permitted in any relevant BMJ Author's Self Archiving Policies, I confirm this Work has not been accepted for publication elsewhere, is not being considered for publication elsewhere and does not duplicate material already published. I confirm all authors consent to publication of this Work and authorise the granting of this licence.

For peer review only

Understanding the importance of non-material factors in retaining community health workers in low-income settings: A qualitative case-study of Ethiopia

Nikita Arora[1*], Kara Hanson[1], Neil Spicer[1], Abiy Seifu Estifanos[2], Dorka Woldesenbet Keraga[2], Alemtsehay Tewele Weleareg ay[3], Freweini Gebrearegay Tela[3], Yemisrach Ahmed Hussen[2], Yordanos Semu Mandefro[2], Matthew Quaife[1]

[1]Department of Global Health and Development, Faculty of Public Health and Policy, London School of Hygiene and Tropical Medicine, UK [2]School of Public Health, Addis Ababa University, Addis Ababa, Ethiopia [3]School of Public Health, Mekelle University, Mekelle, Ethiopia

*corresponding author

Correspondence to:

Nikita Arora, 15-17 Tavistock Pl, Saint Pancras, London WC1H 9SH, Nikita.arora@lshtm.ac.uk, +44 7926991085

Word Count: 4422

For peer review only

ABSTRACT

Objectives

The motivation and retention of community health workers (CHWs) is a challenge and inadequately addressed in research and policy. We sought to identify factors influencing the retention of CHWs in Ethiopia, and ways to mitigate their exit.

Design

A qualitative study was undertaken using in-depth interviews with the study participants. Interviews were audio-recorded, and simultaneously translated into English and transcribed for analysis. Data were analysed in NVivo 12 using an iterative inductive-deductive approach.

Setting

The study was conducted in two regions in Ethiopia: Tigray and Southern Nations, Nationalities, and People's Republic (SNNPR).

Participants

Leavers of HEW positions (n=20), active HEWs (n=16), and key informants (n=11) in the form of policymakers were interviewed.

Results

We identified several extrinsic and intrinsic motivational factors affecting the retention and labour market choices of HEWs. While financial incentives in the form of salaries, and material incentives in the form of improvements in health facility infrastructure and provision of childcare were reported to be important, non-material factors like HEWs' self-image, acceptance and validation by the community and their supervisors were found to be critical. A reduction or loss of these non-material factors proved to be the catalyst for many HEWs to leave their jobs.

Conclusion

Our findings from two regions in Ethiopia contribute new empirical evidence to the global debate on factors influencing the motivation and retention of CHWs, by being the first study to include job leavers in the analysis. Our findings suggest that policy interventions that appeal to the social needs of CHWs could represent a simpler and more cost-effective means of improving their retention. This is

For peer review only

For peer review only - http://bmjopen.bmj.com/site/about/guidelines.xhtml

important for government policymakers in resource constraint settings like Ethiopia that rely heavily on lay workers for primary healthcare delivery.

Strengths and Limitations of the Study

- To the best of our knowledge, this is the first study to report the perspective of leavers of CHW positions, to understand the drivers of their decisions
- While material factors such as salaries and improvements to the health facility were found to be important for the motivation of CHWs in Ethiopia, non-material factors like community acceptance and appreciation were key, and the lack of these led many CHWs to leave their jobs
- An understanding of non-material factors influencing the retention of CHWs is important for policymakers as provision of such factors can be a cost-effective way of retaining these workers, especially in resource constraint settings
- We employed an iterative inductive-deductive style of analyses to allow for relevant themes to be selected, while also allowing unexpected themes to be reflected in participant narratives
- Participants were recruited from within the country, and so a limitation of the study was that we could not capture the perspectives of leavers who had migrated out of Ethiopia

For peer review only - http://bmjopen.bmj.com/site/about/guidelines.xhtml

INTRODUCTION

With 24% of the global burden of disease and only 3% of the global health workforce, countries in Sub-Saharan Africa are struggling to attain universal health coverage and meet sustainable development goals by 2030 (1). In this context, over the last two decades, a large body of evidence has emerged on the importance of Community Health Workers (CHWs) in overcoming workforce shortages and improving population health, particularly in previously underserved communities (2-6). Although the model and scope of CHW programs vary, these health workers are primarily female, trained for a short period on the interventions they will deliver, and usually reside in the communities where they work (7). The significance of CHWs has also been recognised in two recent reports; a World Health Organisation (WHO) guideline on optimising CHW programmes (8) and the CHW Assessment and Improvement Matrix (9) — both of which recommend strategies to optimize the functioning of CHW programmes in health systems, especially in low-and middle-income countries (LMICs) (10).

Although now seen as critical to a well-functioning health system, poor motivation and retention of CHWs remains a challenge. While there is some, limited evidence of effective interventions to address poor motivation and retention (11-14), existing research has typically only explored how different, material incentive packages could improve performance (15-17). Importantly, studies have not explored the role that community culture and group identification play in influencing CHWs' preferences, motivation and retention in the long term. In particular, no study to date has studied CHWs who have left the health system to understand why they left, and what could have mitigated their exit.

In 2003, Ethiopia launched the Health Extension Program (HEP), a primary healthcare delivery strategy designed to make up for the low number of doctors, nurses, and midwives. HEP has focused on delivering essential healthcare services using lay CHWs called Health Extension Workers (HEW), mainly targeting agrarian communities (18). HEWs complete a year-long training in delivering primary healthcare interventions like family planning services, latrine construction, and basic

For peer review only - http://bmjopen.bmj.com/site/about/guidelines.xhtml

preventive and curative services for communicable and non-communicable diseases (19). Unlike CHWs in many other countries, HEWs are salaried government employees with currently more than 40,000 members deployed in the country (18, 20, 21). HEP has recently been recognised by WHO as one of the role models for global CHW programs, due to its focus on integrating CHWs in the health system as civil servants; training HEWs for a significant period of a year before deployment; and offering educational opportunities to upgrade to higher levels of the health workforce (1).

A recent evaluation of HEP found that work satisfaction among HEWs was low, implying poor retention, with high risk of losing a large number of HEWs if and when alternative job opportunities emerge (22). HEWs take up a large proportion of the Ethiopian health budget; 21% of the recurrent health expenditure in 2010/11 was spent on HEW salaries (23), and so it is critical to make sure that experienced HEWs are retained over time to use this budget efficiently but also to sustain the delivery of quality healthcare. Yet, to date, few studies have researched why HEWs leave their posts. Most research has sought to identify financial and non-financial incentives, which motivate HEWs (24-29). Some ethnographic accounts of HEWs have also studied the context in which they work (30-32), and more broadly, research has been conducted on contextual factors influencing the performance of CHWs (26).

While material incentives that align with the preferences of CHWs are relevant to studying retention, empirical findings have seldom been used to theorise their social behaviour, which is tied in with the culture and social context in which they work, thereby influencing their labour choices to leave or stay in their jobs. Moreover, previous studies have never researched the perspective of those who have left these positions (‘leavers’), to capture the drivers of their decisions.

This study identifies factors influencing the labour market decision of CHWs in Ethiopia to leave or stay in their jobs, taking the perspectives of current HEWs, leavers, and the health system. Furthermore, we use the data generated from qualitative interviews to demonstrate how group identification can also influence the social behaviour and preferences of HEWs towards working

For peer review only - http://bmjopen.bmj.com/site/about/guidelines.xhtml

conditions that ultimately influence retention in the health workforce. It is a useful contribution to the global literature on retention of CHWs, as countries gear towards strengthening their own CHW programs.

METHODS

We conducted this study between January and August 2019. In the first stage, we undertook a literature review to identify conceptual frameworks that link motivational factors to health worker retention. We adapted the conceptual framework by Ormel et al, 2019 (33), shown in Figure 1 which critically analyses the use of a mix of incentives and their relationship with CHW motivation and work behaviour, to include the role that pro-social preferences play towards prioritising non-material incentives, highlighted in red in the figure. This model was most in line with our study objectives and thus selected to inform our interview topic guides and categorise our qualitative data described in the section below. Following this, we conducted qualitative interviews with HEWs, leavers, and policy makers.

Figure.1

Study setting

Qualitative in-depth interview data were collected from one district in each of two Ethiopian regions: Tigray and Southern Nations Nationalities and People's Republic (SNNPR), between May and July 2019. Two regions were purposively sampled to capture the varying extent of HEP implementation and thus HEW retention in different regions, which is likely to differ due to the political set up in Ethiopia. Historically, Tigray has been a better performing region on health indicators, in comparison to SNNPR, and so we expected variation in perspectives from staff working in the two regions.

Sampling and participants

Data were collected from three key populations: active HEWs, leavers, and key informants. Key informants were policymakers at the national, regional and district level. Details on sampling and respondents per region are presented in Table 1.

For peer review only - http://bmjopen.bmj.com/site/about/guidelines.xhtml

Table 1: Interviews conducted per informant type

Study population	Active HEWs	Leavers of HEW positions	Key informants
Sample size	16 (8 per region)	20 (10 per region)	11 (5 per region, plus one from Federal Ministry of Health)
Gender	All female	All female	3 female, 8 male
Purpose of inquiry	To capture their perspective on factors affecting HEW motivation and labour choices	To understand factors influencing decisions to leave	To capture the perspective of key stakeholders and identify policy levers that could be modified to improve HEW retention
Sampling technique	Maximum variation sampling - for diversity of age, geographical location and years of experience	Snowball sampling	Purposive sampling, with variation in administrative levels, seniority and level of engagement with HEWs

We conducted 47 semi-structured interviews in total. The mean age of respondents across all three study groups was 31 years, and mean time worked in the health system was 5 years for HEWs, 7 years for key informants and 6 years for leavers. Key informants included HEW supervisors, senior officials at district health offices, HEW experts at district levels, HEW coordinators at regional levels, and a senior official at the HEP directorate, Federal Ministry of Health

Conduct of the interviews

Separate topic guides were drafted for each study population, informed by literature on factors affecting the motivation and labour choices of CHWs in LMICs, and the framework by Ormel et al (33). Key topics covered in the interviews included reasons for choosing the HEW profession, motivating factors, challenges faced in their jobs, and preferences towards job attributes. For leavers, we additionally inquired about their reasons for leaving. Topic guides were piloted and pre-tested with interviewers and members of the study populations. Research Assistants, experienced in

For peer review only - http://bmjopen.bmj.com/site/about/guidelines.xhtml

qualitative research, conducted interviews after receiving two days of orientation on the study aims, topic guides and ethics of research and its conduct. They were all Ethiopian women between the age of 24 and 35 years. Respondents above the age of 18 and willing to participate were approached by Research Assistants through telephone calls. Principles of confidentiality and informed written consent were upheld during interview administration, in compliance with the ethical approval conditions of the project. Each interview was conducted in the language local to that region, in private spaces - normally at a health post - and took on average 45 minutes to complete. All interviews except two were audio-recorded, translated and transcribed in English by interviewers, who also took detailed notes, and discussed daily in debriefing sessions between researchers. During analysis, each interview was assigned a unique alphanumeric identification code, and personally identifiable information was removed from transcripts during coding.

Data analysis

Transcripts from the audio recordings were analysed using an iterative, inductive-deductive approach (34) in NVivo 12 (35). Themes were identified by: (1) reading and re-reading the transcripts and making notes on relevant issues; (2) listing out these issues in the form of a codebook and attaching codes to relevant sections; and (3) writing narrative summaries of relevant themes and sub-themes that emerged most frequently and/or were appropriate to the study (36).

Patient and public involvement

As this study was mainly focussed towards capturing the perspectives of CHWs and policymakers, it was undertaken without the involvement of patients.

RESULTS

Since leavers were the key population of interest in the study, we first describe the jobs that they were engaged in now, and summarize factors that speeded up their exit from HEW positions. Key factors influencing HEW motivation and labour choices are then presented in detail.

For peer review only - http://bmjopen.bmj.com/site/about/guidelines.xhtml

Leavers' destinations

We found significant variation in the type of employment that the leavers were currently engaged in. Migration to non-health jobs in the Middle East, due to better pay was a particularly unanticipated finding. Notably, no participant reported leaving or wanting to leave Ethiopia to work in a health system abroad, perhaps because HEWs are relatively low skilled by international standards.

"Most HEWs think about going to foreign countries. Like Arab countries. The salary in Arab country is relatively good in comparison with HEWs. […] They think, here the workload is very high and the salary is very low so, why don't I go to an Arab country? And why don't I change my life in a short time?"

– *Active HEW, SNNPR*

A significant number of leavers reported to have become full-time homemakers. *"I raise my children, I am a housewife"* said a leaver from Tigray. Some women were currently self-employed and owned small businesses highlighting increased earnings and autonomy. "*I have my own grocery. If you are an excellent worker, it provides higher income. It also has freedom; nobody can come and shout at you"* reported a leaver from Tigray. Other respondents remained working in different health system roles, including as lab technicians and administrators in government health facilities.

Catalysts influencing exit: pro-social preferences

While HEWs did not generally anticipate leaving their jobs, they did leave when they felt they had lost the appreciation of their community or supervisors. These were the two main reasons for leavers to finally quit their jobs, despite other challenging working conditions reported by active HEWs and leavers alike. We call these factors *catalysts,* or the triggers that speeded up the process of attrition:

Failure to receive support and validation from supervisors and senior staff

Conflict with supervisors and senior managers was the main reason why leavers claim to have quit. These 'conflicts' often seem to have started with a senior official disrespecting the HEW, resulting in a

For peer review only - http://bmjopen.bmj.com/site/about/guidelines.xhtml

negative shift in their status, social standing and esteem, and thus in their identity as a HEW. Supportive supervision, with appropriate acknowledgement and validation from their managers was identified as a critical factor in the retention of HEWs in Ethiopia.

"...the director came to my home and insulted me when I was very sick. He said this institution is neither your mother's nor father's; either perform your job appropriately or leave. I immediately left my job, and didn't even take my monthly salary"

-Leaver, Tigray

Reduced acceptance and validation from the community

Another key element for retention was receiving respect, acceptance and validation from the community for whom the HEWs worked. Despite tough working conditions, the opportunity to improve community health attracted many to their jobs. A negative shift in their social identity, due to low community acceptance, influenced working conditions and status, and their exit.

"There is no appreciation from the people in my woreda (district)... always they will criticize the HEW and service delivery... they are fault finders."

-Leaver, SNNPR

Key factors influencing HEW motivation and retention

Numerous factors reported by HEWs, leavers, and KIs were identified as those influencing HEW motivation, and retention in the workforce. Using our conceptual model from Fig 1, we classified these into two categories: Extrinsic and Intrinsic.

Extrinsic motivational factors

Financial incentives

Financial incentives in the form of salaries or wages were found to be important among active HEWs as well as leavers. Current salaries were not considered to be commensurate with workload, their compensation not being enough to cover monthly household expenditure.

For peer review only - http://bmjopen.bmj.com/site/about/guidelines.xhtml

"HEWs do many overlapping tasks, but salary doesn't balance the work we do…the salary does not reflect living conditions of HEWs. Since we don't have additional income, and spend all our time at work, it's difficult to live on existing salary."

- *Active HEW, SNNPR*

Key informants, including HEW supervisors and senior officials at health centers agreed that HEW salaries were inadequate. HEWs cater to a large population, often in topographically difficult terrains and on foot, so physical strain due to their job came up as a common theme and a constraint to their motivation. *"I still remember how horrible it was…the 4 hour walk in the mountains. It rains over us, and the sun burns so bad"*, remembers a leaver from SNNPR.

Material Factors

Material factors were also seen as important in influencing HEW motivation and retention. These often took the form of adequate drugs, equipment and infrastructure available at the health post. Such factors were found to be critical not only to support their daily work, but important to sustain the rapport and faith the community had in them by managing to do the tasks entrusted to them. Sometimes facilities were perceived so lacking that faith was the only answer:

"Sometimes I support the labouring mothers by praying to Gebriel (Angel), because what we learn is different from what we apply. The materials that we have are inadequate; we only have delivery kit, which contains scissor, and cord tie. When a mother delivers at hospital, many things are provided to her and her baby, but here we have nothing to give her".

-Leaver, Tigray

In addition, HEWs and leavers suggested that material incentives such as motorcycles for transportation should be provided as part of their work package, to decrease their physical burden.

Furthermore, the gender of HEWs results in a double burden, as many mothers with infants mentioned that it was hard for them to do their daily tasks as a HEW, alongside caring for their

For peer review only - http://bmjopen.bmj.com/site/about/guidelines.xhtml

infants.

"It is very difficult having a child. I leave from my home early morning at 6 am […] I may stay up to 6 pm, sometimes I don't even have time to drink water after coming back from field work . So, imagine doing all things having a baby"

-Leaver, Tigray

For HEWs with young children, the absence of childcare was a disincentive to continue in their jobs after giving birth.

HEWs also mentioned not always feeling safe in travelling to rural areas. *"Facilities like motor for transportation should be fulfilled. This security issue also needs attention since in rural areas females can be abused,"* stated a leaver from Tigray.

Non-material factors

Most importantly, HEWs and leavers mentioned highly appreciating the non-material factors such as appreciation from their communities and supervisors. The opportunity to improve community health, especially that of mothers and children, and gain their community's trust, respect and acceptance, was unanimously described as the top factor motivating them in their jobs.

"When I get the acceptance of healthy mothers and children, I am satisfied. Otherwise, the salary is not enough; the high workload is as I told you before".

- Active HEW, SNNPR

Sometimes, HEWs were not as easily accepted by their community, which demotivated them. Often respondents claimed that these demand-side barriers existed because of low levels of education and awareness among community members, which also led them to reject healthcare interventions such as family planning and latrine construction.

For peer review only - http://bmjopen.bmj.com/site/about/guidelines.xhtml

"The community's behaviour is difficult. For example, when we go to their home to educate them about environmental hygiene, they may close their door and leave from home. They say, oh! She is coming! When I enter through the front door, they will leave the house from the back door. It is for them but they do not understand. To teach them about something we will take many days. They have a shortage of knowledge."

\- *Leaver, Tigray*

Other non-material demotivating factors were things that HEWs and leavers identified as lacking in HEW jobs. For example, the placement of HEWs in health posts, often far away from their hometowns where their husband and children are based, limited their motivation and retention. All three study populations agreed that the absence of opportunities to transfer to a facility closer to their family was frustrating, unfair, and led HEWs to leave their positions. *"This was my main reason to leave my job… Imagine that you can't meet with your husband as well as your children for a long time because there is no transfer (opportunity)",* mentioned a leaver from Tigray.

In addition, two key issues around career progression were identified. First, HEWs that were keen to upskill to the next level[1] had to take a competitive exam in English – a language they are not generally proficient in and do not normally use in their jobs, and on topics in which they had not received enough training. The success rate for these exams was thus found to be low. HEWs complained that while many of them are excellent field workers with many years of experience in delivering healthcare, their inability to do well in an exam should not be the sole determinant of career progression.

The second key issue was for HEWs who did manage to upgrade to the next level, but despite upskilling, were expected to return to their old jobs at the health post. Many HEWs agreed that while

[1] The majority of HEWs are currently hired as level 3 (now increasingly level 4) health workers and according to HEP, HEWs have the opportunity to upskill to the next level after taking a competitive exam. After this, based on opportunities available and skills needed in the district, HEWs can further upgrade to diploma level courses in subjects like midwifery, and even complete a master's program in public health from government universities.

For peer review only - http://bmjopen.bmj.com/site/about/guidelines.xhtml

after upgrading, their remuneration increased (or was expected to increase in the following months); they were expected to undertake the same tasks, in the same health post as before.

"After we get back from our level 4 study, we will be placed to the same kebele (village) as before. We need to be refreshed, be in a new place! Alongside with transfer, we should also be assigned to health centres (promoted to a higher health facility)",

-Active HEW, SNNPR

Another reason why HEWs and leavers felt de-motivated was the lack of support, oversight, and acknowledgement from supervisors and managers, who said that supervision was based on a model of faultfinding, not mentorship.

"…[…]. I was so tired that night that I could not clean all the blood and every mess (after single-headedly doing a delivery at the health post). Next morning the woreda (district) officials showed up and insulted me without considering what I have been through. It was so painful not to be understood to this level."

- *Leaver, SNNPR*

Intrinsic motivational factors

Many HEWs mentioned that the key reason for joining the profession was to serve the community where they were raised.

"Most of the time in our environment, the mothers don't use contraceptives, they don't give birth in health centres and they don't get ante-natal care. The mothers normally give birth in their home with a traditional birth attendant. Because of this, many mothers die. When I saw these types of problems in my community, I decided to become a HEW. "

- *Active HEW, SNNPR*

Some HEWs also insisted that financial incentives were less important than intrinsic factors and that the profession requires women to be truly dedicated to the community's health improvement, to survive in their jobs.

For peer review only - http://bmjopen.bmj.com/site/about/guidelines.xhtml

Many leavers mentioned having left their jobs out of frustration with challenging conditions, but confessed to have really enjoyed working towards improving community health. *"Regarding the profession, health extension work itself has no concerns. I believe as a HEW you get to serve or work for the community which is great... it's the working conditions that are problematic,"* said a leaver from Tigray.

DISCUSSION

Our study findings from two regions in Ethiopia contribute new empirical evidence to the global knowledge and debate on factors influencing the motivation and retention of CHWs in LMICs, by being the first study to include the perspectives of those who had left the service, in the analysis. Many of the extrinsic motivational factors we identified, such as wages and allowances, were similar to those identified for CHWs in other settings (11, 24, 27, 33, 37, 38). For example, a study in Bangladesh reported lack of time to attend to their own children and other household responsibilities, insufficient profit/salary, and their families' disapproval as reasons cited by CHWs for leaving their posts (39). In Nigeria, village health workers reported low work satisfaction due to the lack of career advancement opportunities, low salaries and poor supervision (40).

Our case study of Ethiopia offers a number of new perspectives that we believe are valuable in Ethiopia and in other LMICs. Discussion around an adequate career path for CHWs in LMICs is ongoing (8, 33, 37). Despite WHO's recommendation for a set career ladder for CHWs to be established in individual country contexts (8), the uptake of this recommendation has been low, as seen in Ethiopia. This was also a key topic of discussion during a recent international symposium on CHWs in 2019 (41).

Other material and non-material incentives affecting retention in this context were better living and working conditions that included their ability to live close to their family, have easy access to water and electricity at home and at work. According to a study published in 2007 (42), the living and working conditions of HEWs during early stages of HEP had not met basic standards. A more recent

For peer review only - http://bmjopen.bmj.com/site/about/guidelines.xhtml

study suggested that many health posts were still missing basic infrastructure like water supply, electricity in 2012 (43). The mean availability of tracer items for basic facilities, infection prevention, malaria diagnosis, and essential medicines at health posts was 37, 29, 52 and 47%, respectively, according to data from a service availability and readiness assessment, in 2016. (44) (45).

Additionally, there is growing recognition for achieving gender inclusiveness and equity in healthcare, which entails transforming the systems within which women work, such as highlighted in a recent report from the World Health Organization's Gender Equity Hub (46). In a Cochrane review, socio-cultural norms that restrict movement of female CHWs and govern acceptable male-female communications were also identified as barriers to doing their jobs successfully (47). In Ethiopia, since majority HEWs are women of reproductive age, providing them with childcare, particularly for the time they are away for fieldwork, could be a good way to make the work environment more inclusive and supportive for these women.

Social behaviour and preferences of HEWs

While our evidence supports the importance of material incentives, we also identified other influences on social preferences of CHWs, which could help understand how they prioritize across multiple factors. Such insights could inform the development of new interventions to motivate and satisfy CHWs and retain them in the long term.

In this context, while conventional models have identified motivation as intrinsic and extrinsic, our empirical results identified two further additions – pro-social preferences as a non-material motivator, and social identity as a factor that could influence how CHWs trade among attributes. The social identity approach demonstrates how processes within an individual that influence behaviour are dependent upon interpersonal relationships and group memberships, as well as their perceived value and significance to the individual (48, 49). This approach states that when a person identifies as a member of a group, and when a given group identity is relevant to an individual; their behaviour

For peer review only - http://bmjopen.bmj.com/site/about/guidelines.xhtml

becomes more focused towards what is seen to be in the group's interest, rather than their own (49, 50).

Thus, when workers define themselves more in terms of personal identity it could be expected that individual motivators such as personal advancement and financial incentives may be more influential. But when defining themselves in terms of social identity, motivators that impact on the group one identifies with, such as their status, standing and acceptance in the group may become more influential (51), like in the case of HEWs. This is a hypothesis that merits further empirical investigation. While the social identity approach is increasingly being applied in high-income countries (11, 51), it is less common in LMICs. To our knowledge, the inSCALE project, which operated in Uganda and Mozambique (11), is the first to use the social identity approach in a LMIC context to address these constraints in motivation of CHWs. Our study is the first to apply the social identity approach for establishing a link between identification and motivation (52, 53) in the context of CHWs in Ethiopia.

CONCLUSION

Our study showed that CHW jobs in LMICs including Ethiopia continue to be challenging, and incentives that align with their preferences have the potential to improve their motivation, influencing retention. However, modifying material incentives alone might not improve retention in the long term. Using empirical data from our study and theories of CHW motivation from the literature, we have demonstrated that CHWs identify themselves as members of a group (in this case their community and team). Thus, appealing to their social needs may represent a relatively simple, cost-effective and complementary strategy to the traditional approach of tailored material incentive packages for improving retention, particularly in resource-constrained settings. This is important for government policymakers in resource constraint settings like Ethiopia that are struggling with critical health workforce shortages and inadequate health budgets. The voices of health workers can offer insights that may otherwise be missed and should thus be included while designing programs to improve retention.

For peer review only - http://bmjopen.bmj.com/site/about/guidelines.xhtml

DECLARATIONS

Ethics approval

Ethical approval was obtained from the London School of Hygiene and Tropical Medicine, UK (ref no. 16177), aswell as Addis Ababa University, Ethiopia (ref. no. 015/19/SPH) in March 2019.

Patient consent for publication

Not required

Data sharing statement

The data sets generated and analysed in the study are available on reasonable request made to the corresponding author.

Competing interests

None declared.

Funding

The Fieldwork for this study was funded by the Wellcome Trust (grant 212771/Z/18/Z), and the IDEAS project, London School of Hygiene and Tropical Medicine.

Author's contributions

All authors were involved in the original design of the qualitative study in Ethiopia. AW, FT, YH, YM conducted and translated all the interviews. DK, AE provided extensive in-country support. NA was the principal investigator who oversaw the fieldwork and conducted majority of the analysis, reviewed and approved by MQ and KH. NS provided expert guidance on manuscript development and analysis. All authors read and approved the final manuscript.

For peer review only

Acknowledgements

The authors would like to thank all the respondents who participated in the interviews.

REFERENCES

1. World Health Organization. Leave no one behind: Strengthening health systems for UHC and the SDGs in Africa. 2017.
2. Bhutta ZA, Lassi ZS, Pariyo G, Huicho LJGhwA. Global experience of community health workers for delivery of health related millennium development goals: a systematic review, country case studies, and recommendations for integration into national health systems. 2010;1(249):61.
3. Lewin S, Dick J, Pond P, Zwarenstein M, Aja GN, van Wyk BE, et al. Lay health workers in primary and community health care for maternal and child health and the management of infectious diseases Cochrane Database Syst Rev. 2005(1).
4. Perry H, Zulliger R. An overview of current evidence with recommendations for strengthening community health worker programs to accelerate progress in achieving the health-related Millennium Development Goals. . Baltimore: Johns Hopkins Bloomberg School of Public Health. 2012:84.
5. Perry HB, Zulliger R, Rogers MM. Community health workers in low-, middle-, and high-income countries: an overview of their history, recent evolution, and current effectiveness. Annual Review of Public Health 2014;35:399-421.
6. Cometto G, Ford N, Pfaffman-Zambruni J, Akl EA, Lehmann U, McPake B, et al. Health policy and system support to optimise community health worker programmes: an abridged WHO guideline. The Lancet Global Health. 2018;6(12):e1397-e404.
7. Lehmann U, Sanders D. Community health workers: what do we know about them. Bulletin of the World Health Organization 2007:1-42.
8. World Health Organization. WHO guideline on health policy and system support to optimize community health worker programmes. 2018.
9. Madeleine Ballard MHB, Jodi-Ann Burey, Jennifer Foth, Kevin Fiori Jr, Isaac Holeman, Ari Johnson, Serah Malaba, Daniel Palazuelos, Mallika Raghavan, Ash Rogers et al. . Community Health Worker Assessment and Improvement Matrix (CHW AIM): Updated Program Functionality Matrix for Optimizing Community Health Programs. 2018.
10. Brown C, Lilford R, Griffiths F, Oppong-Darko P, Ndambo M, Okoh-Owusu M, et al. Case study of a method of development of a selection process for community health workers in sub-Saharan Africa. Human Resources for Health. 2019;17(1):75.
11. Strachan DL, Kallander K, Nakirunda M, Ndima S, Muiambo A, Hill Z, et al. Using theory and formative research to design interventions to improve community health worker motivation, retention and performance in Mozambique and Uganda. Hum Resour Health. 2015;13:25.
12. Bhattacharyya K, Winch P, LeBan K, Tien M. Community health worker incentives and disincentives: how they affect motivation retention and sustainability. 2001.
13. Mueller D, Kurowski C, Mills A. Managing health workforce performance. Component–literature review: determinants and levers of health worker motivation and satisfaction. Health Economics Financing Program London: London School of Hygiene Tropical Medicine International Health. 2005.

For peer review only - http://bmjopen.bmj.com/site/about/guidelines.xhtml

14. Chandler CI, Chonya S, Mtei F, Reyburn H, Whitty CJ. Motivation, money and respect: a mixed-method study of Tanzanian non-physician clinicians. Social science and medicine 2009;68(11):2078-88.
15. Agarwal S, Anaba U, Abuya T, Kintu R, Casseus A, Hossain S, et al. Understanding incentive preferences of community health workers using discrete choice experiments: a multicountry protocol for Kenya, Uganda, Bangladesh and Haiti. BMJ Open. 2019;9(12):e033601.
16. Shiratori S, Agyekum EO, Shibanuma A, Oduro A, Okawa S, Enuameh Y, et al. Motivation and incentive preferences of community health officers in Ghana: an economic behavioral experiment approach. Human resources for health. 2016;14(1):53.
17. Abdel-All M, Angell B, Jan S, Howell M, Howard K, Abimbola S, et al. What do community health workers want? Findings of a discrete choice experiment among Accredited Social Health Activists (ASHAs) in India. 2019;4(3):e001509.
18. Federal Ministry of Health (FMOH). Health Sector Strategic Plan (HSDP-III) 2005/6-2009/10. 2005 2 December 2019.
19. The World Bank. The Health Extension Program in Ethiopia 2012.
20. Federal Ministry of Health E. Health Sector Trsnaformation Plan (HSTP) 2015/16 - 2019/20. 2015.
21. World Bank. Ethiopia health extension program: an institutionalized community approach for universal health coverage 2016.
22. MERQ Consultancy Plc. NATIONAL ASSESSMENT OF THE ETHIOPIAN HEALTH EXTENSION PROGRAM, ABRIDGED REPORT 2019.
23. Wang H, Tesfaye R, NV Ramana G, Chekagn CT. Ethiopia health extension program: an institutionalized community approach for universal health coverage: The World Bank; 2016.
24. Kok MC, Kea AZ, Datiko DG, Broerse JEW, Dieleman M, Taegtmeyer M, et al. A qualitative assessment of health extension workers' relationships with the community and health sector in Ethiopia: opportunities for enhancing maternal health performance. Human Resources for Health. 2015;13(1):80.
25. Kok MC, Ormel H, Broerse JEW, Kane S, Namakhoma I, Otiso L, et al. Optimising the benefits of community health workers' unique position between communities and the health sector: A comparative analysis of factors shaping relationships in four countries. Glob Public Health. 2017;12(11):1404-32.
26. Kok MC, Kane SS, Tulloch O, Ormel H, Theobald S, Dieleman M, et al. How does context influence performance of community health workers in low- and middle-income countries? Evidence from the literature. Health Res Policy Syst. 2015;13:13.
27. Mohammed S, Tilahun M, Kote M, Mama M, Tamiru D. Validation of Health Extension Workers Job Motivation Scale in Gamo-Gofa Zone, Southern Ethiopia: A Cross-Sectional Study. Int Sch Res Notices. 2015;2015:250610.
28. Yayehyirad Kitaw YY-E, Amir Said, Hailay Desta, and Awash Teklehaimanot. Assessment of the Training of the First Intake of Health Extension Workers. Ethiopian Journal of Health Development 2007.
29. Kane S, Kok M, Ormel H, Otiso L, Sidat M, Namakhoma I, et al. Limits and opportunities to community health worker empowerment: A multi-country comparative study. Social Science & Medicine. 2016;164:27-34.
30. Maes KC, Kohrt BA, Closser S. Culture, status and context in community health worker pay: pitfalls and opportunities for policy research. A commentary on Glenton et al. (2010). Social Science & Medicine. 2010;71(8):1375-8; discussion 9-80.

For peer review only

For peer review only - http://bmjopen.bmj.com/site/about/guidelines.xhtml

31. Maes K, Closser S, Kalofonos I. Listening to community health workers: how ethnographic research can inform positive relationships among community health workers, health institutions, and communities. Am J Public Health. 2014;104(5):e5-9.
32. Maes K, Closser S, Tesfaye Y, Gilbert Y, Abesha R. Volunteers in Ethiopia's women's development army are more deprived and distressed than their neighbors: cross-sectional survey data from rural Ethiopia. BMC Public Health. 2018;18(1):258.
33. Ormel H, Kok M, Kane S, Ahmed R, Chikaphupha K, Rashid SF, et al. Salaried and voluntary community health workers: exploring how incentives and expectation gaps influence motivation. Human Resources for Health. 2019;17(1):59.
34. Braun V, Clarke V. Using thematic analysis in psychology. Qualitative Research in Psychology. 2006;3(2):77-101.
35. QSR International Pty Ltd. Version 12. NVivo qualitative data analysis software2018.
36. Takemura T, Kielmann K, Blaauw D. Job preferences among clinical officers in public sector facilities in rural Kenya: a discrete choice experiment. Journal of Human resources for health. 2016;14(1):1.
37. Li L, Hu H, Zhou H, He C, Fan L, Liu X, et al. Work stress, work motivation and their effects on job satisfaction in community health workers: a cross-sectional survey in China. BMJ Open. 2014;4(6):e004897.
38. Chevalier C, Lapo A, O'Brien J, Wierzba TF. Why do village health workers drop out? World Health Forum. 1993;14(3):258-61.
39. Khan SH, Chowdhury AM, Karim F, Barua MK. Training and retaining Shasthyo Shebika: reasons for turnover of community health workers in Bangladesh. Health Care Superv. 1998;17(1):37-47.
40. Gray HH, Ciroma J. Reducing attrition among village health workers in rural Nigeria. Socio-Economic Planning Sciences. 1988;22(1):39-43.
41. International Centre for Diarrhoeal Disease Reserach B. CHW 2019 Symposium [Available from: http://chwsymposium2019.icddrb.org/.
42. Kitaw Y, Ye-Ebiyo Y, Said A, Desta H, Teklehaimanot A. Assessment of the training of the first intake of health extension workers. The Ethiopian Journal of Health Development. 2007;21(3).
43. Medhanyie A, Spigt M, Dinant G, Blanco R. Knowledge and performance of the Ethiopian health extension workers on antenatal and delivery care: a cross-sectional study. Human resources for health. 2012;10(1):44.
44. Ethiopian Public Health Institute (EPHI). Ethiopian Service Availability and Readiness Assessment 2016 Summary Report Addis Ababa, Ethiopia.
45. Assefa Y, Gelaw YA, Hill PS, Taye BW, Van Damme W. Community health extension program of Ethiopia, 2003–2018: successes and challenges toward universal coverage for primary healthcare services. Globalization and Health. 2019;15(1):24.
46. World Health Organization. Delivered by women, led by men: a gender and equity analysis of the global health and social workforce. 2019.
47. Sarin E, Lunsford SS. How female community health workers navigate work challenges and why there are still gaps in their performance: a look at female community health workers in maternal and child health in two Indian districts through a reciprocal determinism framework. Hum Resour Health. 2017;15(1):44.
48. Turner J, Reynolds K. Rediscovering social identity: core sources. The Story of Social Identity. 2010:13-32.
49. Lewis T. Assessing social identity and collective efficacy as theories of group motivation at work. The international journal of human resource management. 2011;22(04):963-80.

For peer review only - http://bmjopen.bmj.com/site/about/guidelines.xhtml

50. Postmes T, Haslam SA, Jans L. A single-item measure of social identification: Reliability, validity, and utility. British journal of social psychology. 2013;52(4):597-617.
51. Haslam SA. Psychology in organizations: Sage; 2004.
52. Van Dick R, Wagner U. Social identification among school teachers: Dimensions, foci, and correlates. European Journal of Work Organizational Psychology. 2002;11(2):129-49.
53. Wegge J, Van Dick R, Fisher GK, Wecking C, Moltzen K. Work motivation, organisational identification, and well-being in call centre work. Journal of Work Stress. 2006;20(1):60-8

For peer review only

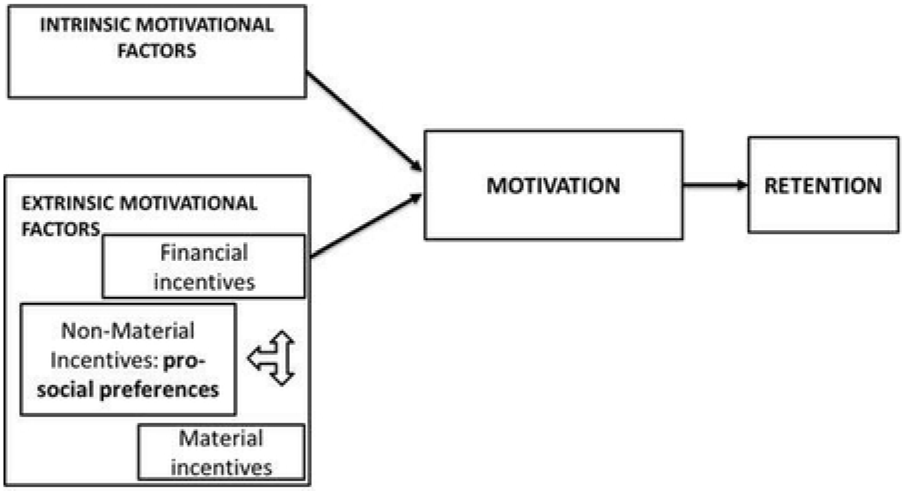

A framework of relationships between motivational factors, motivation and CHW work behaviour. Modified from Ormel et al

38x20mm (300 x 300 DPI)

BMJ Open

Understanding the importance of non-material factors in retaining community health workers in low-income settings: A qualitative case-study in Ethiopia

Journal:	*BMJ Open*
Manuscript ID	bmjopen-2020-037989.R1
Article Type:	Original research
Date Submitted by the Author:	08-Aug-2020
Complete List of Authors:	Arora, Nikita; London School of Hygiene & Tropical Medicine, Department of Global Health and Development<br>Hanson, Kara; London School of Hygiene & Tropical Medicine, Department of Global Health Development<br>Spicer, Neil; London School of Hygiene & Tropical Medicine, Department of Global Health and Development<br>Estifanos, Abiy; Addis Ababa University School of Public Health, School of Public Health<br>Keraga, Dorka; Addis Ababa University, School of Public Health<br>Welearegay, Alemtsehay; Mekelle University, School of Public Health<br>Tela, Freweini; Mekelle University, School of Public Health<br>Hussen, Yemisrach ; Addis Ababa University, School of Public Health<br>Mandefro, Yordanos ; Addis Ababa University, School of Public Health<br>Quaife, Matthew; London School of Hygiene and Tropical Medicine Faculty of Infectious and Tropical Diseases, Faculty of Public Health and Policy
**Primary Subject Heading**:	Health policy
Secondary Subject Heading:	Global health, Health services research, Qualitative research, Public health
Keywords:	Health policy < HEALTH SERVICES ADMINISTRATION & MANAGEMENT, QUALITATIVE RESEARCH, Human resource management < HEALTH SERVICES ADMINISTRATION & MANAGEMENT

SCHOLARONE™
Manuscripts

I, the Submitting Author has the right to grant and does grant on behalf of all authors of the Work (as defined in the below author licence), an exclusive licence and/or a non-exclusive licence for contributions from authors who are: i) UK Crown employees; ii) where BMJ has agreed a CC-BY licence shall apply, and/or iii) in accordance with the terms applicable for US Federal Government officers or employees acting as part of their official duties; on a worldwide, perpetual, irrevocable, royalty-free basis to BMJ Publishing Group Ltd ("BMJ") its licensees and where the relevant Journal is co-owned by BMJ to the co-owners of the Journal, to publish the Work in this journal and any other BMJ products and to exploit all rights, as set out in our licence.

The Submitting Author accepts and understands that any supply made under these terms is made by BMJ to the Submitting Author unless you are acting as an employee on behalf of your employer or a postgraduate student of an affiliated institution which is paying any applicable article publishing charge ("APC") for Open Access articles. Where the Submitting Author wishes to make the Work available on an Open Access basis (and intends to pay the relevant APC), the terms of reuse of such Open Access shall be governed by a Creative Commons licence – details of these licences and which Creative Commons licence will apply to this Work are set out in our licence referred to above.

Other than as permitted in any relevant BMJ Author's Self Archiving Policies, I confirm this Work has not been accepted for publication elsewhere, is not being considered for publication elsewhere and does not duplicate material already published. I confirm all authors consent to publication of this Work and authorise the granting of this licence.

For peer review only

Understanding the importance of non-material factors in retaining community health workers in low-income settings: A qualitative case-study in Ethiopia

Nikita Arora[1*], Kara Hanson[1], Neil Spicer[1], Abiy Seifu Estifanos[2], Dorka Woldesenbet Keraga[2], Alemtsehay Tewele Welearegay[3], Freweini Gebrearegay Tela[3], Yemisrach Ahmed Hussen[2], Yordanos Semu Mandefro[2], Matthew Quaife[1]

[1]Department of Global Health and Development, Faculty of Public Health and Policy, London School of Hygiene and Tropical Medicine, UK [2]School of Public Health, Addis Ababa University, Addis Ababa, Ethiopia [3]School of Public Health, Mekelle University, Mekelle, Ethiopia

*corresponding author

Correspondence to:

Nikita Arora, 15-17 Tavistock Pl, Saint Pancras, London WC1H 9SH,

Nikita.arora@lshtm.ac.uk,

+44 7926991085

Word Count: 5258

For peer review only

ABSTRACT

Objectives

The motivation, retention of community health workers (CHWs) is a challenge and inadequately addressed in research and policy. We sought to identify factors influencing the retention of CHWs in Ethiopia, and ways to mitigate their exit.

Design

A qualitative study was undertaken using in-depth interviews with the study participants. Interviews were audio-recorded, and then simultaneously translated into English and transcribed for analysis. Data were analysed in NVivo 12 using an iterative inductive-deductive approach.

Setting

The study was conducted in two districts, each in the Tigray and Southern Nations, Nationalities, and People's Republic (SNNPR) regions in Ethiopia. Respondents were located in a mix of rural and urban settings.

Participants

Leavers of HEW positions (n=20), active HEWs (n=16), and key informants (n=11) in the form of policymakers were interviewed.

Results

We identified several extrinsic, intrinsic motivational factors affecting the retention and labour market choices of Health Extension Workers (HEWs). While financial incentives in the form of salaries and material incentives in the form of improvements to health facility infrastructure, provision of childcare were reported to be important, non-material factors like HEWs' self-image, acceptance and validation by the community and their supervisors were found to be critical. A reduction or loss of these non-material factors proved to be the catalyst for many HEWs to leave their jobs.

For peer review only - http://bmjopen.bmj.com/site/about/guidelines.xhtml

Conclusion

Our findings from two regions in Ethiopia contribute new empirical evidence to the global debate on factors influencing the motivation and retention of CHWs, by being the first study to include job leavers in the analysis. Our findings suggest that policy interventions that appeal to the social needs of CHWs could represent a simpler and more cost-effective means of improving their retention. This is important for government policymakers in resource constrained settings like Ethiopia that rely heavily on lay workers for primary healthcare delivery.

Strengths and Limitations of the Study

- To the best of our knowledge, this is the first study to report the perspective of leavers of CHW positions, to understand the drivers of their decisions
- We provide an understanding of non-material factors influencing the retention of CHWs, which is important for policymakers to manage attrition among these workers in a cost-effective manner especially in resource constrained settings
- We employed an iterative inductive-deductive style of analyses to allow for relevant themes to be selected, while also allowing unexpected themes to be reflected in participant narratives
- Participants were recruited from within the country so a limitation of the study was our inability to capture the perspectives of leavers who had migrated out of Ethiopia

For peer review only

For peer review only - http://bmjopen.bmj.com/site/about/guidelines.xhtml

INTRODUCTION

With 24% of the global burden of disease and only 3% of the global health workforce, countries in Sub-Saharan Africa are struggling to attain universal health coverage and meet sustainable development goals by 2030 (1). In this context, over the last two decades, a large body of evidence has emerged on the importance of Community Health Workers (CHWs) in overcoming workforce shortages and improving population health, particularly in previously underserved communities (2-6). Although the model and scope of CHW programs vary, these health workers are mostly female, trained for a short period on the interventions they will deliver, and usually reside in communities where they work (7). The significance of CHWs has also been recognised in two recent reports; a World Health Organisation (WHO) guideline on optimising CHW programmes (8) and the CHW Assessment and Improvement Matrix (9) — both of which recommend strategies to optimize the functioning of CHW programmes in health systems, especially in low-and middle-income countries (LMICs) (10). Although now seen as critical to a well-functioning health system, poor motivation and increased attrition amongst CHWs remains a challenge. While there is some, limited evidence of effective interventions to address poor motivation and retention (11-14), existing research has typically only explored how different, material incentive packages could improve performance (15-17). Importantly, studies have not explored the role that community culture and group identification play in influencing CHWs' preferences, motivation and retention in the long term. In particular, no study to date has studied CHWs who have left the health system to understand why they left, and what could have mitigated their exit.

In 2003, Ethiopia launched the Health Extension Program (HEP), a primary healthcare delivery strategy designed to make up for the low number of doctors, nurses, and midwives. HEP has focused on delivering essential healthcare services using lay CHWs called Health Extension Workers (HEW), mainly targeting agrarian communities (18). HEWs complete a year-long training in delivering primary healthcare interventions like family planning services,

For peer review only - http://bmjopen.bmj.com/site/about/guidelines.xhtml

latrine construction, and basic preventive and curative services for communicable and non-communicable diseases (19). Unlike CHWs in many other countries, HEWs are salaried government employees with currently more than 40,000 members deployed in the country (18, 20, 21). HEP has recently been recognised by WHO as a role model for global CHW programs, due to its focus on integrating CHWs in the health system as civil servants; training HEWs for a significant period of a year before deployment; and offering educational opportunities to upgrade to higher levels of the health workforce (8).

A recent, national evaluation of HEP had found the overall job satisfaction of HEWs to be quite low. More than half of the study sample reported to be unsatisfied with their current posts, suggesting that their retention could be affected in the long run. These apprehensions were substantiated by data indicating a gradual rise in the rate of attrition amongst HEWs over the programmatic lifetime of HEP, between 2005 and 2019. The average annual rate of attrition was reported to be close to 3%, with overall attrition since the start of the program being 21%. (22) This showed a clear rise in HEW attrition since the last national assessment of HEP published in 2011, which estimated overall attrition in the cadre for the period between 2005 and 2010 to be 6.5% (23).

HEWs take up a large proportion of the Ethiopian health budget; 21% of the recurrent health expenditure in 2010/11 was spent on HEW salaries (21), and so it is critical to make sure that experienced HEWs are retained over time to use this budget efficiently but also to sustain the delivery of quality healthcare. Yet, to date, few studies have researched why HEWs leave their posts. Most research has sought to identify financial and non-financial incentives, which motivate HEWs (24-29). Some ethnographic accounts of HEWs have also studied the context in which they work (30-32), and more broadly, research has been conducted on contextual factors influencing the performance of CHWs (26).

For peer review only - http://bmjopen.bmj.com/site/about/guidelines.xhtml

While material incentives that align with the preferences of CHWs are relevant to studying retention, behavioural theories like the social identity approach have seldom been applied to empirical findings, to account for the social behaviour of health workers. This approach studies the social identity, the context in which they work, and how certain factors could influence their labour choices to leave or stay in their jobs. Moreover, previous studies have never researched the perspective of CHWs who have left these positions ('leavers'), to capture the drivers of their decisions.

This study identifies factors influencing the labour market decision of CHWs in Ethiopia to leave or stay in their jobs, taking the perspectives of current HEWs, leavers, and the health system. Furthermore, we use the data generated from qualitative interviews to demonstrate how group identification can also influence the social behaviour and preferences of HEWs towards working conditions that ultimately influence retention in the health workforce. This evidence makes an original contribution to the global literature on retention of CHWs, as countries gear towards strengthening their own CHW programs.

METHODS

We conducted this study between January and August 2019. In the first stage, we undertook a literature review to identify conceptual frameworks that link motivational factors to health worker retention. We adapted the conceptual framework by Ormel et al, 2019 (33), shown in Figure 1 which critically analyses the use of a mix of incentives and their relationship with CHW motivation and work behaviour, to include the role that pro-social preferences play towards prioritising non-material incentives, highlighted in red in the figure. This model was most in line with our study objectives and thus selected to inform our interview topic guides and categorise our qualitative data described in the section below. Following this, we conducted qualitative narrative research with HEWs, leavers, and policy makers.

For peer review only - http://bmjopen.bmj.com/site/about/guidelines.xhtml

For peer review only

Study setting

Qualitative in-depth interview data were collected from two districts, each in two Ethiopian regions: Tigray and Southern Nations Nationalities and People's Republic (SNNPR), between May and July 2019. Raya Azebo, Saharti Samra districts were covered in Tigray and Dilla Zuria, Silte in SNNPR. HEWs were located in a mix of urban and rural backgrounds. The regions were purposively sampled to capture the varying extent of HEP implementation and thus HEW retention in different regions, which was likely to differ due to the political set up in Ethiopia. Historically, Tigray has been a better performing region on health indicators, in comparison to SNNPR, and so we expected variation in perspectives from staff working in the two regions.

Sampling and participants

Data were collected from three key populations: active HEWs, leavers, and key informants. Key informants were policymakers at the national, regional and district level. Details on sampling and respondents per region are presented in Table 1.

Table 1: Interviews conducted per informant type

Study population	Active HEWs	Leavers of HEW positions	Key informants
Sample size	16 (8 per region)	20 (10 per region)	11 (5 per region, plus one from Federal Ministry of Health)
Gender	All females	All females	3 females, 8 males
Marital status	10 married, 4 single, 2 divorced	8 married, 1 divorced, 1 single	10 married, 1 single
Purpose of inquiry	To capture their perspective on factors affecting HEW motivation and labour choices	To understand factors influencing decisions to leave	To capture the perspective of key stakeholders and identify policy levers that could be modified to improve HEW retention

For peer review only - http://bmjopen.bmj.com/site/about/guidelines.xhtml

Sampling technique	Maximum variation sampling from a list of HEWs working in study districts, for diversity of age, geographical location and years of experience	Snowball sampling	Purposive sampling, with variation in administrative levels, seniority and level of engagement with HEWs

We interviewed respondents until sufficient saturation was attained in the themes emerging from interview data. A total of 47 semi-structured interviews were conducted. The mean age of respondents across all three study groups was 31 years, ranging from 24 to 40 for leavers, 20 to 49 for HEWs and 26 to 48 for KIs. The mean time worked in the health system was 6 years for HEWs, ranging between 1 and 13 years and 7 years for key informants, ranging from 0.5 to 8 years. Leavers had spent on average 6 years in their current jobs, ranging from 1 to 9 years. Key informants included HEW supervisors, senior officials at district health offices, HEW experts at district levels, HEW coordinators at regional levels, and a senior official at the HEP directorate, Federal Ministry of Health.

Conduct of the interviews

Separate topic guides were drafted for each study population, informed by literature on factors affecting the motivation and labour choices of CHWs in LMICs, and the framework by Ormel et al (33). Key topics covered in the interviews included reasons for choosing the HEW profession, motivating factors, challenges faced in their jobs, and preferences towards job attributes. For leavers, we additionally inquired about their reasons for leaving. Topic guides were piloted and pre-tested with interviewers and members of the study populations. Research assistants experienced in qualitative research conducted interviews after receiving two days' training on study aims, topic guides, ethics of research and its conduct. Training included how to identify and reduce social desirability tendencies in respondents. All research assistants were Ethiopian women between ages 24 and 35 years. Respondents above the age of 18 and willing to participate were approached by research assistants

For peer review only

For peer review only - http://bmjopen.bmj.com/site/about/guidelines.xhtml

through telephone calls. Principles of confidentiality and informed written consent were upheld during interview administration, in compliance with the ethical approval conditions of the project. Each interview was conducted in the language local to that region, in private spaces - normally at the back side of the health post where the respondent and interviewer could be left alone. As much as possible, the research assistants held interviews when HEWs were comparatively less busy with work, and took on average 45 minutes to complete. All interviews except two were audio-recorded, translated and transcribed in English by interviewers, who also took notes and discussed in daily debriefing sessions between researchers. For the two interviews where audio recording was not possible due to respondent refusal, research assistants took down detailed notes which were later used for analysis. Each interview was assigned a unique alphanumeric identification code during analysis, and personally identifiable information was removed from transcripts during coding.

Data analysis

Transcripts from the audio recordings were analyzed using an iterative, inductive-deductive approach (34) in NVivo 12 (35). Themes were identified by: (1) reading and re-reading the transcripts and making notes on relevant issues; (2) listing out these issues in the form of a codebook and attaching codes to relevant sections; and (3) writing narrative summaries of relevant themes and sub-themes that emerged most frequently and/or were appropriate to the study (36).

Patient and public involvement

This study focussed on capturing the perspectives of CHWs and policymakers, and was undertaken without the involvement of patients.

RESULTS

Since leavers were the key population of interest in the study, we first describe the jobs that they were engaged at the time of interview and summarize factors that speeded up their exit

For peer review only - http://bmjopen.bmj.com/site/about/guidelines.xhtml

from HEW positions. Key factors influencing HEW motivation and labour choices are then presented in detail.

Leavers' destinations

We found significant variation in the type of employment that the leavers were currently engaged in. Migration to non-health jobs in the Middle East, due to better pay was a particularly unanticipated finding. Notably, no participant reported leaving or wanting to leave Ethiopia to work in a health system abroad, perhaps because HEWs are relatively low skilled by international standards.

"Most HEWs think about going to foreign countries. Like Arab countries. The salary in Arab country is relatively good in comparison with HEWs. […] They think, here the workload is very high and the salary is very low so, why don't I go to an Arab country? And why don't I change my life in a short time?"

– *Active HEW, SNNPR*

A significant number of leavers reported to have become full-time homemakers. *"I raise my children, I am a housewife"* said a leaver from Tigray. Some women were currently self-employed and owned small businesses highlighting increased earnings and autonomy. "*I have my own grocery. If you are an excellent worker, it provides higher income. It also has freedom; nobody can come and shout at you"* reported a leaver from Tigray. Other respondents remained working in different health system roles, including as lab technicians and administrators in government health facilities.

Catalysts influencing exit: pro-social preferences

While HEWs did not generally anticipate leaving their jobs, they did leave when they felt they had lost the appreciation of their community or supervisors. These were the two main reasons for leavers to finally quit their jobs, despite other challenging working conditions

For peer review only - http://bmjopen.bmj.com/site/about/guidelines.xhtml

For peer review only

reported by active HEWs and leavers alike. We call these factors *catalysts,* or the triggers that speeded up the process of attrition:

Failure to receive support and validation from supervisors and senior staff

Conflict with supervisors and senior managers was the main reason why leavers claim to have quit. These ‘conflicts’ often seem to have started with a senior official disrespecting the HEW, resulting in a negative shift in their status, social standing and esteem, and thus in their identity as a HEW. Supportive supervision, with appropriate acknowledgement and validation from their managers was identified as a critical factor in the retention of HEWs in Ethiopia.

“…the director came to my home and insulted me when I was very sick. He said this institution is neither your mother’s nor father’s; either perform your job appropriately or leave. I immediately left my job, and didn’t even take my monthly salary”

-Leaver, Tigray

Reduced acceptance and validation from the community

Another key element for retention was receiving respect, acceptance and validation from the community for whom the HEWs worked. Despite tough working conditions, the opportunity to improve community health attracted many to their jobs. A negative shift in their social identity, due to low community acceptance, influenced working conditions and status, and their exit.

“There is no appreciation from the people in my woreda (district)… always they will criticize the HEW and service delivery… they are fault finders.”

-Leaver, SNNPR

For peer review only - http://bmjopen.bmj.com/site/about/guidelines.xhtml

Key factors influencing HEW motivation and retention

Numerous factors reported by HEWs, leavers, and KIs were identified as those influencing HEW motivation, and retention in the workforce. Using our conceptual model from Fig 1, we classified these into two categories: Extrinsic and Intrinsic.

Extrinsic motivational factors

Financial incentives

Financial incentives in the form of salaries or wages were found to be important among active HEWs as well as leavers. Current salaries were not considered to be commensurate with workload, their compensation not being enough to cover monthly household expenditure.

"HEWs do many overlapping tasks, but salary doesn't balance the work we do…the salary does not reflect living conditions of HEWs. Since we don't have additional income, and spend all our time at work, it's difficult to live on existing salary."

- *Active HEW, SNNPR*

Key informants, including HEW supervisors and senior officials at health centers unanimously agreed that HEW salaries were inadequate. HEWs cater to a large population, often in topographically difficult terrains and on foot, so physical strain due to their job came up as a common theme and a constraint to their motivation. *"I still remember how horrible it was…the 4 hour walk in the mountains. It rains over us, and the sun burns so bad"*, remembers a leaver from SNNPR.

Material Factors

Material factors were also seen as important in influencing HEW motivation and retention. These often took the form of adequate drugs, equipment and infrastructure available at the health post. Such factors were found to be critical not only to support their daily work, but important to sustain the rapport and faith the community had in them by managing to do the

For peer review only - http://bmjopen.bmj.com/site/about/guidelines.xhtml

tasks entrusted to them. Sometimes facilities were perceived so lacking that faith was the only answer:

“Sometimes I support the labouring mothers by praying to Gebriel (Angel), because what we learn is different from what we apply. The materials that we have are inadequate; we only have delivery kit, which contains scissor, and cord tie. When a mother delivers at hospital, many things are provided to her and her baby, but here we have nothing to give her”.

-Leaver, Tigray

In addition, HEWs and leavers suggested that material incentives such as motorcycles for transportation should be provided as part of their work package, to decrease their physical burden.

Furthermore, the gender of HEWs results in a double burden, as many mothers with infants mentioned that it was hard for them to do their daily tasks as a HEW, alongside caring for their infants.

“It is very difficult having a child. I leave from my home early morning at 6 am […] I may stay up to 6 pm, sometimes I don’t even have time to drink water after coming back from field work . So, imagine doing all things having a baby”

-Leaver, Tigray

For HEWs with young children, the absence of childcare was a disincentive to continue in their jobs after giving birth.

HEWs also mentioned not always feeling safe in travelling to rural areas. *“Facilities like motor for transportation should be fulfilled. This security issue also needs attention since in rural areas females can be abused,”* stated a leaver from Tigray.

For peer review only - http://bmjopen.bmj.com/site/about/guidelines.xhtml

Non-material factors

Most importantly, HEWs and leavers mentioned highly appreciating the non-material factors such as appreciation from their communities and supervisors. The opportunity to improve community health, especially that of mothers and children, and gain their community's trust, respect and acceptance, was unanimously described as the top factor motivating them in their jobs.

"*When I get the acceptance of healthy mothers and children, I am satisfied. Otherwise, the salary is not enough; the high workload is as I told you before".*

- Active HEW, SNNPR

Sometimes, HEWs were not as easily accepted by their community, which demotivated them. Often respondents claimed that these demand-side barriers existed because of low levels of education and awareness among community members, which also led them to reject healthcare interventions such as family planning and latrine construction.

"The community's behaviour is difficult. For example, when we go to their home to educate them about environmental hygiene, they may close their door and leave from home. They say, oh! She is coming! When I enter through the front door, they will leave the house from the back door. It is for them but they do not understand. To teach them about something we will take many days. They have a shortage of knowledge."

- Leaver, Tigray

Other non-material demotivating factors were things that HEWs and leavers identified as lacking in HEW jobs. For example, the placement of HEWs in health posts, often far away from their hometowns where their husband and children are based, limited their motivation and retention. All three study populations agreed that the absence of opportunities to transfer to a facility closer to their family was frustrating, unfair, and led HEWs to leave their positions. *"This was my main reason to leave my job… Imagine that you can't meet with*

For peer review only - http://bmjopen.bmj.com/site/about/guidelines.xhtml

For peer review only

your husband as well as your children for a long time because there is no transfer (opportunity)”, mentioned a leaver from Tigray.

In addition, two key issues around career progression were identified. First, HEWs that were keen to upskill to the next level[1] had to take a competitive exam in English – a language they are not generally proficient in and do not normally use in their jobs, and on topics in which they had not received enough training. The success rate for these exams was thus found to be low. HEWs complained that while many of them are excellent field workers with many years of experience in delivering healthcare, their inability to do well in an exam should not be the sole determinant of career progression.

The second key issue was for HEWs who did manage to upgrade to the next level, but despite upskilling, were expected to return to their old jobs at the health post. Many HEWs agreed that while after upgrading, their remuneration increased (or was expected to increase in the following months); they were expected to undertake the same tasks, in the same health post as before.

“After we get back from our level 4 study, we will be placed to the same kebele (village) as before. We need to be refreshed, be in a new place! Alongside with transfer, we should also be assigned to health centres (promoted to a higher health facility)”,

-Active HEW, SNNPR

Another reason why HEWs and leavers felt de-motivated was the lack of support, oversight, and acknowledgement from supervisors and managers, who said that supervision was based on a model of faultfinding, not mentorship.

[1] The majority of HEWs are currently hired as level 3 (now increasingly level 4) health workers and according to HEP, HEWs have the opportunity to upskill to the next level after taking a competitive exam. After this, based on opportunities available and skills needed in the district, HEWs can further upgrade to diploma level courses in subjects like midwifery, and even complete a master’s program in public health from government universities.

For peer review only - http://bmjopen.bmj.com/site/about/guidelines.xhtml

"…[…]. I was so tired that night that I could not clean all the blood and every mess (after single- headedly doing a delivery at the health post). Next morning the woreda (district) officials showed up and insulted me without considering what I have been through. It was so painful not to be understood to this level."

- *Leaver, SNNPR*

Intrinsic motivational factors

Many HEWs mentioned that the key reason for joining the profession was to serve the community where they were raised.

"Most of the time in our environment, the mothers don't use contraceptives, they don't give birth in health centres and they don't get ante-natal care. The mothers normally give birth in their home with a traditional birth attendant. Because of this, many mothers die. When I saw these types of problems in my community, I decided to become a HEW. "

- *Active HEW, SNNPR*

Some HEWs also insisted that financial incentives were less important than intrinsic factors and that the profession requires women to be truly dedicated to the community's health improvement, to survive in their jobs.

Many leavers mentioned having left their jobs out of frustration with challenging conditions but confessed to have really enjoyed working towards improving community health. *"Regarding the profession, health extension work itself has no concerns. I believe as a HEW you get to serve or work for the community which is great... it's the working conditions that are problematic,"* said a leaver from Tigray.

DISCUSSION

Our study findings from two regions in Ethiopia contribute new empirical evidence to the global knowledge and debate on factors influencing the motivation and retention of CHWs in LMICs. It is the first study to include the perspectives of those who had left their posts. Since we wanted to capture the individual experiences and behaviour of all three respondent

For peer review only - http://bmjopen.bmj.com/site/about/guidelines.xhtml

groups, we refrained from conducting focus group discussions and stuck to using in-depth interviews. Moreover, we believe that reasons why people leave their jobs or things they find unsatisfying are of sensitive nature, unlikely to be disclosed in a group of peers.

Many of the extrinsic motivational factors we identified, such as wages and allowances, were similar to those identified for CHWs in other settings (11, 24, 27, 33, 37, 38). For example, a study in Bangladesh reported lack of time to attend to their own children and other household responsibilities, insufficient profit/salary, and their families' disapproval as reasons cited by CHWs for leaving their posts (39). In Nigeria, village health workers reported low work satisfaction due to the lack of career advancement opportunities, low salaries and poor supervision (40).

Our study offers a number of new perspectives that we believe are valuable in Ethiopia and in other LMICs. Discussion around an adequate career path for CHWs in LMICs is ongoing (8, 33, 37). Despite WHO's repeated recommendations on a set career ladder for CHWs to be established in individual country contexts (8), the uptake has been low by governments. For example, in Ethiopia, there is evidence that the majority of HEWs are keen to take on more responsibility and upgrade to become nurses, pharmacy technicians and health administrators (41) but no such career path is offered to them. Access to and provision of upgrading and promotion opportunities was identified as one of top five measures to motivate HEWs and improve HEP services in another study (42). The need for and the value of career progression amongst CHWs to improve job satisfaction, was also a key topic of discussion at an international symposium on CHWs in 2019 (43). Moreover, the rate of attrition was reported to be nearly three times greater for higher level professionals like doctors and nurses, in comparison to other allied health professionals lower in the hierarchy. Factors identified as contributing towards these attrition rates were higher budget constraints, but also the lack of educational opportunities and poor career development (23).

For peer review only - http://bmjopen.bmj.com/site/about/guidelines.xhtml

Other material and non-material incentives affecting retention in this context were better living and working conditions that included their ability to live close to their family, have easy access to water and electricity at home and at work. According to a study published in 2007 (28), the living and working conditions of HEWs during early stages of HEP had not met basic standards. A more recent study suggested that many health posts were still missing basic infrastructure like water supply, electricity in 2012 (44). The mean availability of tracer items for basic facilities, infection prevention, malaria diagnosis, and essential medicines at health posts was 37, 29, 52 and 47%, respectively, according to data from a service availability and readiness assessment, in 2016. (45, 46)

Additionally, there is growing recognition of the importance of gender inclusiveness and equity in healthcare, which entails transforming the systems within which women work, such as highlighted in a recent report from the World Health Organization's Gender Equity Hub (47). In a Cochrane review, socio-cultural norms that restrict movement of female CHWs and govern acceptable male-female communications were also identified as barriers to doing their jobs successfully (48). Jackson et al. (49) apply a gender lens to HEP and to the role of HEWs and conclude that by changing gender norms and reducing constraints to gender equality, HEP could have more transformative outcomes not just for HEWs but for the communities they serve. Since the majority of HEWs in Ethiopia are women of reproductive age, providing them with childcare, particularly for when they are away for house visits, could be a step forward in gender transforming their work environment.

Social behaviour and preferences of HEWs

While our evidence supports the importance of material incentives, we also identified other influences on social preferences of CHWs, which could help understand how they prioritize across multiple factors. Such insights could inform the development of new interventions to motivate and satisfy CHWs and retain them in the long term.

For peer review only - http://bmjopen.bmj.com/site/about/guidelines.xhtml

In this context, while conventional models have identified motivation as intrinsic and extrinsic, our empirical results identified two further additions – pro-social preferences as a non-material motivator, and social identity as a factor that could influence how CHWs trade among attributes. The social identity approach demonstrates how processes within an individual that influence behaviour are dependent upon interpersonal relationships and group memberships, as well as their perceived value and significance to the individual (50, 51). This approach states that when a person identifies as a member of a group, and when a given group identity is relevant to an individual; their behaviour becomes more focused towards what is seen to be in the group's interest, rather than their own (51, 52).

Thus, when workers define themselves in terms of a personal identity it could be expected that individual motivators such as personal advancement and financial incentives may be more influential. However, when defining themselves in terms of a social identity, motivators that impact on the group one identifies with, such as their status, standing and acceptance in the group may become more influential (53), like in the case of HEWs. This is a hypothesis that merits further empirical investigation. While the social identity approach is increasingly being applied in high-income countries (11, 53), it is less common in LMICs. To our knowledge, the inSCALE project, which operated in Uganda and Mozambique (11), is the first to use the social identity approach in a LMIC context to address these constraints in motivation of CHWs. Our study drew on formative research results from the inSCALE study and applied the social identity approach for establishing links between identification and motivation (54, 55) in the context of CHWs in Ethiopia.

It has already been recognised that non-material interventions to support CHWs can contribute substantially to creating a more satisfied health workforce that is able and willing to continue delivering quality healthcare to communities.(31, 49, 56, 57). In the Ethiopian context, focus particularly could be accorded to improve not just the availability of strategic resources such as mentoring and supervision, but the quality of support offered by often male supervisors to these female workers. Addressing HEW aspirations to progress in their

For peer review only - http://bmjopen.bmj.com/site/about/guidelines.xhtml

jobs by providing sufficient upgrading opportunities, tailored to their preferences and abilities, has good potential for improving their job satisfaction, reducing attrition. Clearly, positive community attitude towards HEWs is a key demand-side requirement for HEWs to stay motivated. We believe a good rapport between HEWs and the community often results when HEWs are capable of providing healthcare to the standards expected by the community. This of course is a function of having health posts with adequate infrastructure, well trained HEWs, but equally importantly emotionally satisfied HEWs who have not had to live apart from their families due to lack of transfer opportunities.

Future research should explore the development of interventions that can create and maintain trust between CHWs and the community. It could further be evaluated if a bottom-up approach that is designed with the inputs of CHWs and the community, is better tailored to the needs and realities of both.(25) In addition to health outcomes, policymakers should also invest in studying outcome measures such as competencies and self-esteem of health workers as this can have direct effects on their retention and indirect effects on the sustainable delivery of population health.

CONCLUSION

Our study showed that CHW jobs in LMICs including Ethiopia continue to be challenging, and incentives that align with their preferences have the potential to improve their motivation, influencing retention. However, modifying material incentives alone might not improve retention in the long term. Using empirical data from our study and theories of CHW motivation from the literature, we have demonstrated that CHWs identify themselves as members of a group (in this case their community and team). Thus, appealing to their social needs may represent a relatively simpler, cost-effective and complementary strategy to the traditional approach of using financial incentive packages for improving retention, particularly in resource-constrained settings. These non-material factors are important to be considered

For peer review only - http://bmjopen.bmj.com/site/about/guidelines.xhtml

by government policymakers in resource constrained settings like Ethiopia that are struggling with critical health workforce shortages and inadequate health budgets. The voices of health workers can offer insights that may otherwise be missed and should thus be included while designing programs to improve retention.

DECLARATIONS

Ethics approval

Ethical approval was obtained from the London School of Hygiene and Tropical Medicine, UK (ref no. 16177), aswell as Addis Ababa University, Ethiopia (ref. no. 015/19/SPH) in March 2019.

Patient consent for publication

Not required

Data sharing statement

The data sets generated and analysed in the study are available on reasonable request made to the corresponding author.

Competing interests

None declared.

Funding

The Fieldwork for this study was funded by the Wellcome Trust (grant 212771/Z/18/Z), and the IDEAS project, London School of Hygiene and Tropical Medicine.

Author's contributions

All authors were involved in the original design of the qualitative study in Ethiopia. AW, FT, YH, YM conducted and translated all the interviews. DK, AE provided extensive in-country

For peer review only - http://bmjopen.bmj.com/site/about/guidelines.xhtml

For peer review only

support. NA was the principal investigator who oversaw the fieldwork and conducted majority of the analysis, reviewed and approved by MQ and KH. NS provided expert guidance on manuscript development and analysis. All authors read and approved the final manuscript.

Acknowledgements

The authors would like to thank all the respondents who participated in the interviews.

REFERENCES

1. World Health Organization. Leave no one behind: Strengthening health systems for UHC and the SDGs in Africa. 2017.
2. Bhutta ZA, Lassi ZS, Pariyo G, Huicho L. Global experience of community health workers for delivery of health related millennium development goals: a systematic review, country case studies, and recommendations for integration into national health systems. Global health workforce Alliance. 2010;1(249):61.
3. Lewin S, Dick J, Pond P, Zwarenstein M, Aja GN, van Wyk BE, et al. Lay health workers in primary and community health care for maternal and child health and the management of infectious diseases Cochrane Database Syst Rev. 2005(1).
4. Perry H, Zulliger R. An overview of current evidence with recommendations for strengthening community health worker programs to accelerate progress in achieving the health-related Millennium Development Goals. . Baltimore: Johns Hopkins Bloomberg School of Public Health. 2012:84.
5. Perry HB, Zulliger R, Rogers MM. Community health workers in low-, middle-, and high-income countries: an overview of their history, recent evolution, and current effectiveness. Annual Review of Public Health 2014;35:399-421.
6. Cometto G, Ford N, Pfaffman-Zambruni J, Akl EA, Lehmann U, McPake B, et al. Health policy and system support to optimise community health worker programmes: an abridged WHO guideline. The Lancet Global Health. 2018;6(12):e1397-e404.
7. Lehmann U, Sanders D. Community health workers: what do we know about them. Bulletin of the World Health Organization 2007:1-42.
8. World Health Organization. WHO guideline on health policy and system support to optimize community health worker programmes. 2018.
9. Madeleine Ballard MHB, Jodi-Ann Burey, Jennifer Foth, Kevin Fiori Jr, Isaac Holeman, Ari Johnson, Serah Malaba, Daniel Palazuelos, Mallika Raghavan, Ash Rogers et al. . Community Health Worker Assessment and Improvement Matrix (CHW AIM): Updated Program Functionality Matrix for Optimizing Community Health Programs. 2018.
10. Brown C, Lilford R, Griffiths F, Oppong-Darko P, Ndambo M, Okoh-Owusu M, et al. Case study of a method of development of a selection process for community health workers in sub-Saharan Africa. Human Resources for Health. 2019;17(1):75.

For peer review only

For peer review only - http://bmjopen.bmj.com/site/about/guidelines.xhtml

11. Strachan DL, Källander K, Nakirunda M, Ndima S, Muiambo A, Hill Z. Using theory and formative research to design interventions to improve community health worker motivation, retention and performance in Mozambique and Uganda. Human resources for health. 2015;13(1):25.
12. Bhattacharyya K, Winch P, LeBan K, Tien M. Community health worker incentives and disincentives: how they affect motivation retention and sustainability. 2001.
13. Mueller D, Kurowski C, Mills A. Managing health workforce performance. Component–literature review: determinants and levers of health worker motivation and satisfaction. Health Economics Financing Program London: London School of Hygiene Tropical Medicine International Health. 2005.
14. Chandler CI, Chonya S, Mtei F, Reyburn H, Whitty CJ. Motivation, money and respect: a mixed-method study of Tanzanian non-physician clinicians. Social science and medicine 2009;68(11):2078-88.
15. Agarwal S, Anaba U, Abuya T, Kintu R, Casseus A, Hossain S, et al. Understanding incentive preferences of community health workers using discrete choice experiments: a multicountry protocol for Kenya, Uganda, Bangladesh and Haiti. BMJ Open. 2019;9(12):e033601.
16. Shiratori S, Agyekum EO, Shibanuma A, Oduro A, Okawa S, Enuameh Y, et al. Motivation and incentive preferences of community health officers in Ghana: an economic behavioral experiment approach. Human resources for health. 2016;14(1):53.
17. Abdel-All M, Angell B, Jan S, Howell M, Howard K, Abimbola S, et al. What do community health workers want? Findings of a discrete choice experiment among Accredited Social Health Activists (ASHAs) in India. BMJ Glob Health. 2019;4(3):e001509-e.
18. Federal Ministry of Health (FMOH). Health Sector Strategic Plan (HSDP-III) 2005/6-2009/10. 2005 2 December 2019.
19. The World Bank. The Health Extension Program in Ethiopia 2012.
20. Federal Ministry of Health E. Health Sector Trsnaformation Plan (HSTP) 2015/16 - 2019/20. 2015.
21. Wang H, Tesfaye R, NV Ramana G, Chekagn CT. Ethiopia health extension program: an institutionalized community approach for universal health coverage: The World Bank; 2016.
22. MERQ Consultancy Plc. NATIONAL ASSESSMENT OF THE ETHIOPIAN HEALTH EXTENSION PROGRAM, ABRIDGED REPORT 2019.
23. Hailemichael Y, Jira C, Girma B, Tushune K. Health workforce deployment, attrition and density in East wollega zone, Western ethiopia. Ethiop. 2010;20(1):15-23.
24. Kok MC, Kea AZ, Datiko DG, Broerse JE, Dieleman M, Taegtmeyer M, et al. A qualitative assessment of health extension workers' relationships with the community and health sector in Ethiopia: opportunities for enhancing maternal health performance. Hum Resour Health. 2015;13:80.
25. Kok MC, Ormel H, Broerse JEW, Kane S, Namakhoma I, Otiso L, et al. Optimising the benefits of community health workers' unique position between communities and the health sector: A comparative analysis of factors shaping relationships in four countries. Glob Public Health. 2017;12(11):1404-32.
26. Kok MC, Kane SS, Tulloch O, Ormel H, Theobald S, Dieleman M, et al. How does context influence performance of community health workers in low- and middle-income countries? Evidence from the literature. Health Res Policy Syst. 2015;13:13.

For peer review only - http://bmjopen.bmj.com/site/about/guidelines.xhtml

27. Mohammed S, Tilahun M, Kote M, Mama M, Tamiru D. Validation of Health Extension Workers Job Motivation Scale in Gamo-Gofa Zone, Southern Ethiopia: A Cross-Sectional Study. Int Sch Res Notices. 2015;2015:250610.
28. Kitaw Y, Ye-Ebiyo Y, Said A, Desta H, Teklehaimanot A. Assessment of the training of the first intake of health extension workers. The Ethiopian Journal of Health Development. 2007;21(3).
29. Kane S, Kok M, Ormel H, Otiso L, Sidat M, Namakhoma I, et al. Limits and opportunities to community health worker empowerment: A multi-country comparative study. Social Science & Medicine. 2016;164:27-34.
30. Maes KC, Kohrt BA, Closser S. Culture, status and context in community health worker pay: pitfalls and opportunities for policy research. A commentary on Glenton et al. (2010). Social Science & Medicine. 2010;71(8):1375-8; discussion 9-80.
31. Maes K, Closser S, Kalofonos I. Listening to community health workers: how ethnographic research can inform positive relationships among community health workers, health institutions, and communities. Am J Public Health. 2014;104(5):e5-9.
32. Maes K, Closser S, Tesfaye Y, Gilbert Y, Abesha R. Volunteers in Ethiopia's women's development army are more deprived and distressed than their neighbors: cross-sectional survey data from rural Ethiopia. BMC Public Health. 2018;18(1):258.
33. Ormel H, Kok M, Kane S, Ahmed R, Chikaphupha K, Rashid SF, et al. Salaried and voluntary community health workers: exploring how incentives and expectation gaps influence motivation. Human Resources for Health. 2019;17(1):59.
34. Braun V, Clarke V. Using thematic analysis in psychology. Qualitative Research in Psychology. 2006;3(2):77-101.
35. QSR International Pty Ltd. Version 12. NVivo qualitative data analysis software2018.
36. Takemura T, Kielmann K, Blaauw D. Job preferences among clinical officers in public sector facilities in rural Kenya: a discrete choice experiment. Journal of Human resources for health. 2016;14(1):1.
37. Li L, Hu H, Zhou H, He C, Fan L, Liu X, et al. Work stress, work motivation and their effects on job satisfaction in community health workers: a cross-sectional survey in China. BMJ Open. 2014;4(6):e004897.
38. Chevalier C, Lapo A, O'Brien J, Wierzba TF. Why do village health workers drop out? World Health Forum. 1993;14(3):258-61.
39. Khan SH, Chowdhury A, Karim F, Barua MKJTHcs. Training and retaining Shasthyo Shebika: reasons for turnover of community health workers in Bangladesh. 1998;17(1):37-47.
40. Gray HH, Ciroma J. Reducing attrition among village health workers in rural Nigeria. Socio-Economic Planning Sciences. 1988;22(1):39-43.
41. Awash Teklehaimanot YK, Samuel Girma, Aklilu Seyoum, Yemane Ye-Ebiyo. Study of the working conditions of Health Extension Woekrs in Ethiopia. Ethiopian Journal of Health Development. 2007.
42. Centre for National Health Development in Ethiopia and Columbia University. Ethiopia Health Extension Program Evaluation Study, 2007-2010. Health post and HEWs performance survey. 2011.
43. International Centre for Diarrhoeal Disease Reserach B. CHW 2019 Symposium [Available from: http://chwsymposium2019.icddrb.org/.

For peer review only - http://bmjopen.bmj.com/site/about/guidelines.xhtml

44. Medhanyie A, Spigt M, Dinant G, Blanco R. Knowledge and performance of the Ethiopian health extension workers on antenatal and delivery care: a cross-sectional study. Human resources for health. 2012;10(1):44.

45. Ethiopian Public Health Institute (EPHI). Ethiopian Service Availability and Readiness Assessment 2016 Summary Report Addis Ababa, Ethiopia.

46. Assefa Y, Gelaw YA, Hill PS, Taye BW, Van Damme W. Community health extension program of Ethiopia, 2003–2018: successes and challenges toward universal coverage for primary healthcare services. Globalization and Health. 2019;15(1):24.

47. World Health Organization. Delivered by women, led by men: a gender and equity analysis of the global health and social workforce. 2019.

48. Sarin E, Lunsford SS. How female community health workers navigate work challenges and why there are still gaps in their performance: a look at female community health workers in maternal and child health in two Indian districts through a reciprocal determinism framework. Hum Resour Health. 2017;15(1):44.

49. Jackson R, Kilsby D, Hailemariam A. Gender exploitative and gender transformative aspects of employing Health Extension Workers under Ethiopia's Health Extension Program. Tropical Medicine & International Health. 2019;24(3):304-19.

50. Turner J, Reynolds K. Rediscovering social identity: core sources. The Story of Social Identity. 2010:13-32.

51. Lewis T. Assessing social identity and collective efficacy as theories of group motivation at work. The international journal of human resource management. 2011;22(04):963-80.

52. Postmes T, Haslam SA, Jans L. A single-item measure of social identification: Reliability, validity, and utility. British journal of social psychology. 2013;52(4):597-617.

53. Haslam SA. Psychology in organizations: Sage; 2004.

54. Van Dick R, Wagner U. Social identification among school teachers: Dimensions, foci, and correlates. European Journal of Work Organizational Psychology. 2002;11(2):129-49.

55. Wegge J, Van Dick R, Fisher GK, Wecking C, Moltzen K. Work motivation, organisational identification, and well-being in call centre work. Journal of Work Stress. 2006;20(1):60-83.

56. Jackson R, Hailemariam A. The Role of Health Extension Workers in Linking Pregnant Women With Health Facilities for Delivery in Rural and Pastoralist Areas of Ethiopia. Ethiop. 2016;26(5):471-8.

57. Saran I, Winn L, Kipkoech Kirui J, Menya D, Prudhomme O'Meara W. The relative importance of material and non-material incentives for community health workers: Evidence from a discrete choice experiment in Western Kenya. Social Science & Medicine. 2020;246:112726.

Figure 1: A framework of relationships between motivational factors, motivation and CHW work behaviour. Modified from Ormel et al

For peer review only

For peer review only - http://bmjopen.bmj.com/site/about/guidelines.xhtml

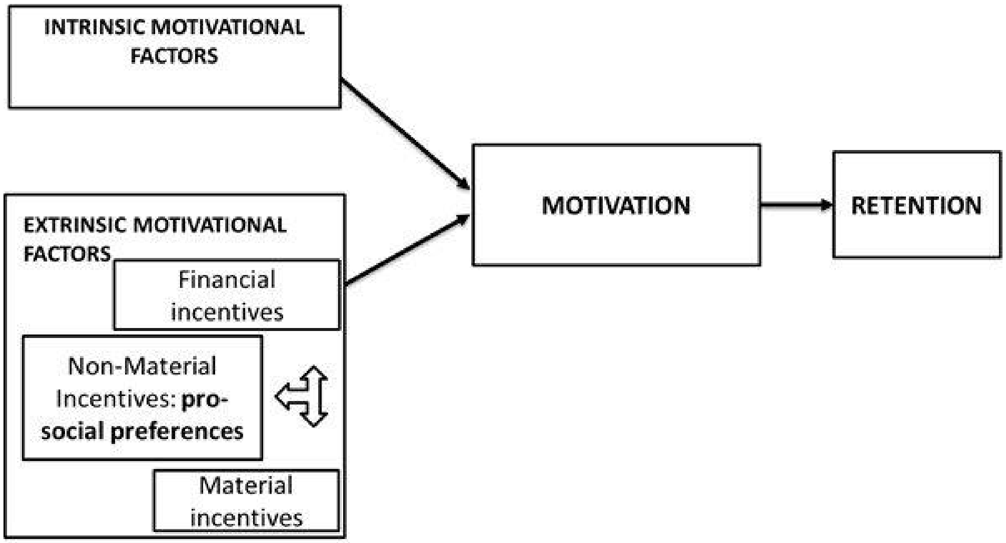

A framework of relationships between motivational factors, motivation and CHW work behaviour. Modified from Ormel et al

38x20mm (400 x 400 DPI)

Standards for Reporting Qualitative Research (SRQR)*

http://www.equator-network.org/reporting-guidelines/srqr/

Title and abstract

	Page/line no(s).
**Title** - Concise description of the nature and topic of the study Identifying the study as qualitative or indicating the approach (e.g., ethnography, grounded theory) or data collection methods (e.g., interview, focus group) is recommended	1/1-3
**Abstract** - Summary of key elements of the study using the abstract format of the intended publication; typically includes background, purpose, methods, results, and conclusions	2-3/28-60

Introduction

**Problem formulation** - Description and significance of the problem/phenomenon studied; review of relevant theory and empirical work; problem statement	4-5/81-134
**Purpose or research question** - Purpose of the study and specific objectives or questions	6/135-149

Methods

**Qualitative approach and research paradigm** - Qualitative approach (e.g., ethnography, grounded theory, case study, phenomenology, narrative research) and guiding theory if appropriate; identifying the research paradigm (e.g., postpositivist, constructivist/ interpretivist) is also recommended; rationale**	6/151-159
**Researcher characteristics and reflexivity** - Researchers' characteristics that may influence the research, including personal attributes, qualifications/experience, relationship with participants, assumptions, and/or presuppositions; potential or actual interaction between researchers' characteristics and the research questions, approach, methods, results, and/or transferability	8, 9/190-212
**Context** - Setting/site and salient contextual factors; rationale**	7/163-171
**Sampling strategy** - How and why research participants, documents, or events were selected; criteria for deciding when no further sampling was necessary (e.g., sampling saturation); rationale**	7/table 1 and lines 172-175
**Ethical issues pertaining to human subjects** - Documentation of approval by an appropriate ethics review board and participant consent, or explanation for lack thereof; other confidentiality and data security issues	Page 21/ 520-522 and page 8,9/190-207
**Data collection methods** - Types of data collected; details of data collection procedures including (as appropriate) start and stop dates of data collection and analysis, iterative process, triangulation of sources/methods, and modification of procedures in response to evolving study findings; rationale**	Page 6 line 151, page 8 lines 196-212, page 9 215-220-211

For peer review only - http://bmjopen.bmj.com/site/about/guidelines.xhtml

**Data collection instruments and technologies** - Description of instruments (e.g., interview guides, questionnaires) and devices (e.g., audio recorders) used for data collection; if/how the instrument(s) changed over the course of the study	Page 8/194-197
**Units of study** - Number and relevant characteristics of participants, documents, or events included in the study; level of participation (could be reported in results)	Page 7, 8/173-187
**Data processing** - Methods for processing data prior to and during analysis, including transcription, data entry, data management and security, verification of data integrity, data coding, and anonymization/de-identification of excerpts	Page 9 /207-212
**Data analysis** - Process by which inferences, themes, etc., were identified and developed, including the researchers involved in data analysis; usually references a specific paradigm or approach; rationale**	Page 9/ 215-220
**Techniques to enhance trustworthiness** - Techniques to enhance trustworthiness and credibility of data analysis (e.g., member checking, audit trail, triangulation); rationale**	Page 9/ 216-220

Results/findings

**Synthesis and interpretation** - Main findings (e.g., interpretations, inferences, and themes); might include development of a theory or model, or integration with prior research or theory	Pages 9-16/225-399
**Links to empirical data** - Evidence (e.g., quotes, field notes, text excerpts, photographs) to substantiate analytic findings	Pages 9-16/225-399

Discussion

**Integration with prior work, implications, transferability, and contribution(s) to the field** - Short summary of main findings; explanation of how findings and conclusions connect to, support, elaborate on, or challenge conclusions of earlier scholarship; discussion of scope of application/generalizability; identification of unique contribution(s) to scholarship in a discipline or field	Page 16 -20/ 401-502
**Limitations** - Trustworthiness and limitations of findings	Page 3/ 61-71

Other

**Conflicts of interest** - Potential sources of influence or perceived influence on study conduct and conclusions; how these were managed	Page21/532
**Funding** - Sources of funding and other support; role of funders in data collection, interpretation, and reporting	Page 21/535-536

*The authors created the SRQR by searching the literature to identify guidelines, reporting standards, and critical appraisal criteria for qualitative research; reviewing the reference lists of retrieved sources; and contacting experts to gain feedback. The SRQR aims to improve the transparency of all aspects of qualitative research by providing clear standards for reporting qualitative research.

For peer review only - http://bmjopen.bmj.com/site/about/guidelines.xhtml

For peer review only

**The rationale should briefly discuss the justification for choosing that theory, approach, method, or technique rather than other options available, the assumptions and limitations implicit in those choices, and how those choices influence study conclusions and transferability. As appropriate, the rationale for several items might be discussed together.

Reference:

O'Brien BC, Harris IB, Beckman TJ, Reed DA, Cook DA. **Standards for reporting qualitative research: a synthesis of recommendations.** *Academic Medicine*, Vol. 89, No. 9 / Sept 2014 DOI: 10.1097/ACM.0000000000000388

For peer review only

For peer review only - http://bmjopen.bmj.com/site/about/guidelines.xhtml

BMJ Open

Understanding the importance of non-material factors in retaining community health workers in low-income settings: A qualitative case-study in Ethiopia

Journal:	*BMJ Open*
Manuscript ID	bmjopen-2020-037989.R2
Article Type:	Original research
Date Submitted by the Author:	28-Aug-2020
Complete List of Authors:	Arora, Nikita; London School of Hygiene & Tropical Medicine, Department of Global Health and Development<br>Hanson, Kara; London School of Hygiene & Tropical Medicine, Department of Global Health Development<br>Spicer, Neil; London School of Hygiene & Tropical Medicine, Department of Global Health and Development<br>Estifanos, Abiy; Addis Ababa University School of Public Health, School of Public Health<br>Keraga, Dorka; Addis Ababa University, School of Public Health<br>Welearegay, Alemtsehay; Mekelle University, School of Public Health<br>Tela, Freweini; Mekelle University, School of Public Health<br>Hussen, Yemisrach ; Addis Ababa University, School of Public Health<br>Mandefro, Yordanos ; Addis Ababa University, School of Public Health<br>Quaife, Matthew; London School of Hygiene and Tropical Medicine Faculty of Infectious and Tropical Diseases, Faculty of Public Health and Policy
**Primary Subject Heading**:	Health policy
Secondary Subject Heading:	Global health, Health services research, Qualitative research, Public health
Keywords:	Health policy < HEALTH SERVICES ADMINISTRATION & MANAGEMENT, QUALITATIVE RESEARCH, Human resource management < HEALTH SERVICES ADMINISTRATION & MANAGEMENT

SCHOLARONE™
Manuscripts

I, the Submitting Author has the right to grant and does grant on behalf of all authors of the Work (as defined in the below author licence), an exclusive licence and/or a non-exclusive licence for contributions from authors who are: i) UK Crown employees; ii) where BMJ has agreed a CC-BY licence shall apply, and/or iii) in accordance with the terms applicable for US Federal Government officers or employees acting as part of their official duties; on a worldwide, perpetual, irrevocable, royalty-free basis to BMJ Publishing Group Ltd ("BMJ") its licensees and where the relevant Journal is co-owned by BMJ to the co-owners of the Journal, to publish the Work in this journal and any other BMJ products and to exploit all rights, as set out in our licence.

The Submitting Author accepts and understands that any supply made under these terms is made by BMJ to the Submitting Author unless you are acting as an employee on behalf of your employer or a postgraduate student of an affiliated institution which is paying any applicable article publishing charge ("APC") for Open Access articles. Where the Submitting Author wishes to make the Work available on an Open Access basis (and intends to pay the relevant APC), the terms of reuse of such Open Access shall be governed by a Creative Commons licence – details of these licences and which Creative Commons licence will apply to this Work are set out in our licence referred to above.

Other than as permitted in any relevant BMJ Author's Self Archiving Policies, I confirm this Work has not been accepted for publication elsewhere, is not being considered for publication elsewhere and does not duplicate material already published. I confirm all authors consent to publication of this Work and authorise the granting of this licence.

For peer review only

Understanding the importance of non-material factors in retaining community health workers in low-income settings: A qualitative case-study in Ethiopia

Nikita Arora[1*], Kara Hanson[1], Neil Spicer[1], Abiy Seifu Estifanos[2], Dorka Woldesenbet Keraga[2], Alemtsehay Tewele Welearegay[3], Freweini Gebrearegay Tela[3], Yemisrach Ahmed Hussen[2], Yordanos Semu Mandefro[2], Matthew Quaife[1]

[1]Department of Global Health and Development, Faculty of Public Health and Policy, London School of Hygiene and Tropical Medicine, UK [2]School of Public Health, Addis Ababa University, Addis Ababa, Ethiopia [3]School of Public Health, Mekelle University, Mekelle, Ethiopia

*corresponding author

Correspondence to:

Nikita Arora, 15-17 Tavistock Pl, Saint Pancras, London WC1H 9SH,

Nikita.arora@lshtm.ac.uk,

+44 7926991085

Word Count: 5235

For peer review only

ABSTRACT

Objectives

The motivation, retention of community health workers (CHWs) is a challenge and inadequately addressed in research and policy. We sought to identify factors influencing the retention of CHWs in Ethiopia, and ways to mitigate their exit.

Design

A qualitative study was undertaken using in-depth interviews with the study participants. Interviews were audio-recorded, and then simultaneously translated into English and transcribed for analysis. Data were analysed in NVivo 12 using an iterative inductive-deductive approach.

Setting

The study was conducted in two districts each in the Tigray and Southern Nations, Nationalities, and People's Republic (SNNPR) regions in Ethiopia. Respondents were located in a mix of rural and urban settings.

Participants

Leavers of Health Extension Worker (HEW) positions (n=20), active HEWs (n=16), and key informants (n=11) in the form of policymakers were interviewed.

Results

We identified several extrinsic, intrinsic motivational factors affecting the retention and labour market choices of HEWs. While financial incentives in the form of salaries and material incentives in the form of improvements to health facility infrastructure, provision of childcare were reported to be important, non-material factors like HEWs' self-image, acceptance and validation by the community and their supervisors were found to be critical. A reduction or loss of these non-material factors proved to be the catalyst for many HEWs to leave their jobs.

For peer review only - http://bmjopen.bmj.com/site/about/guidelines.xhtml

Conclusion

Our study contributes new empirical evidence to the global debate on factors influencing the motivation and retention of CHWs, by being the first to include job leavers in the analysis. Our findings suggest that policy interventions that appeal to the social needs of CHWs can prove to be more acceptable and potentially cost-effective in improving their retention in the long run. This is important for government policymakers in resource constrained settings like Ethiopia that rely heavily on lay workers for primary healthcare delivery.

Strengths and Limitations of the Study

- To the best of our knowledge, this is the first study to report the perspective of leavers of CHW positions, to understand the drivers of their decisions
- We provide an understanding of non-material factors influencing the retention of CHWs, which is important for policymakers to manage attrition among these workers in a cost-effective manner especially in resource constrained settings
- We employed an iterative inductive-deductive style of analyses to allow for relevant themes to be selected, while also allowing unexpected themes to be reflected in participant narratives
- Participants were recruited from within the country so a limitation of the study was our inability to capture the perspectives of leavers who had migrated out of Ethiopia

For peer review only

For peer review only - http://bmjopen.bmj.com/site/about/guidelines.xhtml

INTRODUCTION

With 24% of the global burden of disease and only 3% of the global health workforce, countries in Sub-Saharan Africa are struggling to attain universal health coverage and meet sustainable development goals by 2030 (1). In this context, over the last two decades, a large body of evidence has emerged on the importance of Community Health Workers (CHWs) in overcoming workforce shortages and improving population health, particularly in previously underserved communities (2-6). Although the model and scope of CHW programs vary, these health workers are mostly female, trained for a short period on the interventions they will deliver, and usually reside in communities where they work (7). The significance of CHWs has also been recognised in two recent reports; a World Health Organisation (WHO) guideline on optimising CHW programmes (8) and the CHW Assessment and Improvement Matrix (9) — both of which recommend strategies to optimize the functioning of CHW programmes in health systems, especially in low-and middle-income countries (LMICs) (10). Although now seen as critical to a well-functioning health system, poor motivation and increased attrition amongst CHWs remains a challenge. While there is some limited evidence of effective interventions to address poor motivation and retention (11-14), existing research has typically only explored how different, material incentive packages could improve performance (15-17). Importantly, studies have not explored the role that community culture and group identification play in influencing CHWs' preferences, motivation and retention in the long term. In particular, no study to date has studied CHWs who have left the health system to understand why they left, and what could have mitigated their exit.

In 2003, Ethiopia launched the Health Extension Program (HEP), a primary healthcare delivery strategy designed to make up for the low number of doctors, nurses, and midwives. HEP has focused on delivering essential healthcare services using lay CHWs called Health Extension Workers (HEW), mainly targeting agrarian communities (18). HEWs complete a year-long training in delivering primary healthcare interventions like family planning services,

For peer review only - http://bmjopen.bmj.com/site/about/guidelines.xhtml

For peer review only

latrine construction, and basic preventive and curative services for communicable and non-communicable diseases (19). Unlike CHWs in many other countries, HEWs are salaried government employees with currently more than 40,000 members deployed in the country (18, 20, 21). HEP has recently been recognised by WHO as a role model for global CHW programs, due to its focus on integrating CHWs in the health system as civil servants; training HEWs for a significant period of a year before deployment; and offering educational opportunities to upgrade to higher levels of the health workforce (8).

A recent, national evaluation of HEP had found the overall job satisfaction of HEWs to be quite low. More than half of the study sample reported to be unsatisfied with their current posts, suggesting that their retention could be affected in the long run. These apprehensions were substantiated by data indicating a gradual rise in the rate of attrition amongst HEWs over the programmatic lifetime of HEP, between 2005 and 2019. The average annual rate of attrition was reported to be close to 3%, with overall attrition since the start of the program being 21%. (22) This showed a clear rise in HEW attrition since the last national assessment of HEP published in 2011, which estimated overall attrition in the cadre for the period between 2005 and 2010 to be 6.5% (23).

HEWs take up a large proportion of the Ethiopian health budget; 21% of the recurrent health expenditure in 2010/11 was spent on HEW salaries (21), and so it is critical to make sure that experienced HEWs are retained over time to use this budget efficiently but also to sustain the delivery of quality healthcare. Yet, to date few studies have researched why HEWs leave their posts. Most research has sought to identify financial and non-financial incentives, which motivate HEWs (24-29). Some ethnographic accounts of HEWs have also studied the context in which they work (30-32), and more broadly, research has been conducted on contextual factors influencing the performance of CHWs (26).

For peer review only

For peer review only - http://bmjopen.bmj.com/site/about/guidelines.xhtml

While material incentives that align with the preferences of CHWs are relevant to studying retention, behavioural theories like the social identity approach have seldom been applied to empirical findings, to account for the social behaviour of health workers. This approach studies the social identity, context in which they work, along with self-efficacy and outcome expectancies that could influence their labour choices to stay in or leave their jobs. Further description of this approach is provided in the discussion section. Moreover, previous studies have never researched the perspective of CHWs who have left these positions ('leavers'), to capture the drivers of their decisions.

This study identifies factors influencing the labour market decision of CHWs in Ethiopia to leave or stay in their jobs, taking the perspectives of current HEWs, leavers, and the health system. Furthermore, we use the data generated from qualitative interviews to demonstrate how group identification can also influence the social behaviour and preferences of HEWs towards working conditions that ultimately influence retention in the health workforce. This evidence makes an original contribution to the global literature on retention of CHWs, as countries gear towards strengthening their own CHW programs.

METHODS

We conducted this study between January and August 2019. In the first stage, we undertook a literature review to identify conceptual frameworks that link motivational factors to health worker retention. We adapted the conceptual framework by Ormel et al, 2019 (33), shown in Figure 1 which critically analyses the use of a mix of incentives and their relationship with CHW motivation and work behaviour, to include the role that pro-social preferences play towards prioritising non-material incentives, highlighted in red in the figure. This model was most in line with our study objectives and thus selected to inform our interview topic guides and categorise our qualitative data described in the section below. Following this, we conducted qualitative narrative research with HEWs, leavers, and policy makers.

For peer review only - http://bmjopen.bmj.com/site/about/guidelines.xhtml

162

163 **Study setting**

164 Qualitative in-depth interview data were collected from two districts, each in two Ethiopian
165 regions: Tigray and Southern Nations Nationalities and People's Republic (SNNPR),
166 between May and July 2019. Raya Azebo, Saharti Samra districts were covered in Tigray
167 and Dilla Zuria, Silte in SNNPR. HEWs were located in a mix of urban and rural
168 backgrounds. The regions were purposively sampled to capture the varying extent of HEP
169 implementation and thus HEW retention in different regions, which was likely to differ due to
170 the political set up in Ethiopia. Historically, Tigray has been a better performing region on
171 health indicators, in comparison to SNNPR, and so we expected variation in perspectives
172 from staff working in the two regions.

173 **Sampling and participants**

174 Data were collected from three key populations: active HEWs, leavers, and key informants.
175 Key informants were policymakers at the national, regional and district level. Details on
176 sampling and respondents per region are presented in Table 1.

177

178 **Table 1: Interviews conducted per informant type**

Study population	Active HEWs	Leavers of HEW positions	Key informants
Sample size	16 (8 per region)	20 (10 per region)	11 (5 per region, plus one from Federal Ministry of Health)
Gender	All females	All females	3 females, 8 males
Marital status	10 married, 4 single, 2 divorced	8 married, 1 divorced, 1 single	10 married, 1 single
Purpose of inquiry	To capture their perspective on factors affecting HEW	To understand factors influencing	To capture the perspective of key stakeholders and identify policy levers that

For peer review only - http://bmjopen.bmj.com/site/about/guidelines.xhtml

	motivation and labour choices	decisions to leave	could be modified to improve HEW retention
Sampling technique	Maximum variation sampling from a list of HEWs working in study districts, for diversity of age, geographical location and years of experience	Snowball sampling	Purposive sampling, with variation in administrative levels, seniority and level of engagement with HEWs

We interviewed respondents until sufficient saturation was attained in the themes emerging from interview data. A total of 47 semi-structured interviews were conducted. The mean age of respondents across all three study groups was 31 years, ranging from 24 to 40 for leavers, 20 to 49 for HEWs and 26 to 48 for KIs. The mean time worked in the health system was 6 years for HEWs, ranging between 1 and 13 years and 7 years for key informants, ranging from 0.5 to 8 years. Leavers had spent on average 6 years in their current jobs, ranging from 1 to 9 years. Key informants included HEW supervisors, senior officials at district health offices, HEW experts at district levels, HEW coordinators at regional levels, and a senior official at the HEP directorate, Federal Ministry of Health.

Conduct of the interviews

Separate topic guides were drafted for each study population, informed by literature on factors affecting the motivation and labour choices of CHWs in LMICs, and the framework by Ormel et al (33). Key topics covered in the interviews included reasons for choosing the HEW profession, motivating factors, challenges faced in their jobs, and preferences towards job attributes. For leavers, we additionally inquired about their reasons for leaving. Topic guides were piloted and pre-tested with interviewers and members of the study populations. Research assistants experienced in qualitative research conducted interviews after receiving two days' training on study aims, topic guides, ethics of research and its conduct. Training included how to identify and reduce social desirability tendencies in respondents. All research assistants were Ethiopian women between ages 24 and 35 years. Respondents

For peer review only

For peer review only - http://bmjopen.bmj.com/site/about/guidelines.xhtml

above the age of 18 and willing to participate were approached by research assistants through telephone calls. Principles of confidentiality and informed written consent were upheld during interview administration, in compliance with the ethical approval conditions of the project. Each interview was conducted in the language local to that region, in private spaces - normally at the back side of the health post where the respondent and interviewer could be left alone. As much as possible, the research assistants held interviews when HEWs were comparatively less busy with work, and took on average 45 minutes to complete. All interviews except two were audio-recorded, translated and transcribed in English by interviewers, who also took notes and discussed in daily debriefing sessions between researchers. For the two interviews where audio recording was not possible due to respondent refusal, research assistants took down detailed notes which were later used for analysis. Each interview was assigned a unique alphanumeric identification code during analysis, and personally identifiable information was removed from transcripts during coding.

Data analysis

Transcripts from the audio recordings were analyzed using an iterative, inductive-deductive approach (34) in NVivo 12 (35). Themes were identified by: (1) reading and re-reading the transcripts and making notes on relevant issues; (2) listing out these issues in the form of a codebook and attaching codes to relevant sections; and (3) writing narrative summaries of relevant themes and sub-themes that emerged most frequently and/or were appropriate to the study (36).

Patient and public involvement

This study focussed on capturing the perspectives of CHWs and policymakers, and was undertaken without the involvement of patients.

RESULTS

Since leavers were the key population of interest in the study, we first describe the jobs that they were engaged at the time of interview and summarize factors that speeded up their exit

For peer review only - http://bmjopen.bmj.com/site/about/guidelines.xhtml

from HEW positions. Key factors influencing HEW motivation and labour choices are then presented in detail.

Leavers' destinations

We found significant variation in the type of employment that the leavers were currently engaged in. Migration to non-health jobs in the Middle East, due to better pay was a particularly unanticipated finding. Notably, no participant reported leaving or wanting to leave Ethiopia to work in a health system abroad, perhaps because HEWs are relatively low skilled by international standards.

"Most HEWs think about going to foreign countries. Like Arab countries. The salary in Arab country is relatively good in comparison with HEWs. […] They think, here the workload is very high and the salary is very low so, why don't I go to an Arab country? And why don't I change my life in a short time?"

– *Active HEW, SNNPR*

A significant number of leavers reported to have become full-time homemakers. *"I raise my children, I am a housewife"* said a leaver from Tigray. Some women were currently self-employed and owned small businesses highlighting increased earnings and autonomy. *"I have my own grocery. If you are an excellent worker, it provides higher income. It also has freedom; nobody can come and shout at you"* reported a leaver from Tigray. Other respondents remained working in different health system roles, including as lab technicians and administrators in government health facilities.

Catalysts influencing exit: pro-social preferences

While HEWs did not generally anticipate leaving their jobs, they did leave when they felt they had lost the appreciation of their community or supervisors. These were the two main reasons for leavers to finally quit their jobs, despite other challenging working conditions

For peer review only - http://bmjopen.bmj.com/site/about/guidelines.xhtml

reported by active HEWs and leavers alike. We call these factors *catalysts,* or the triggers that speeded up the process of attrition:

Failure to receive support and validation from supervisors and senior staff

Conflict with supervisors and senior managers was the main reason why leavers claim to have quit. These 'conflicts' often seem to have started with a senior official disrespecting the HEW, resulting in a negative shift in their status, social standing and esteem, and thus in their identity as a HEW. Supportive supervision, with appropriate acknowledgement and validation from their managers was identified as a critical factor in the retention of HEWs in Ethiopia.

"…the director came to my home and insulted me when I was very sick. He said this institution is neither your mother's nor father's; either perform your job appropriately or leave. I immediately left my job, and didn't even take my monthly salary"

-Leaver, Tigray

Reduced acceptance and validation from the community

Another key element for retention was receiving respect, acceptance and validation from the community for whom the HEWs worked. Despite tough working conditions, the opportunity to improve community health attracted many to their jobs. A negative shift in their social identity, due to low community acceptance, influenced working conditions and status, and their exit.

"There is no appreciation from the people in my woreda (district)… always they will criticize the HEW and service delivery… they are fault finders."

-Leaver, SNNPR

For peer review only - http://bmjopen.bmj.com/site/about/guidelines.xhtml

Key factors influencing HEW motivation and retention

Numerous factors reported by HEWs, leavers, and KIs were identified as those influencing HEW motivation, and retention in the workforce. Using our conceptual model from Fig 1, we classified these into two categories: Extrinsic and Intrinsic.

Extrinsic motivational factors

Financial incentives

Financial incentives in the form of salaries or wages were found to be important among active HEWs as well as leavers. Current salaries were not considered to be commensurate with workload, their compensation not being enough to cover monthly household expenditure.

"HEWs do many overlapping tasks, but salary doesn't balance the work we do…the salary does not reflect living conditions of HEWs. Since we don't have additional income, and spend all our time at work, it's difficult to live on existing salary."

\- *Active HEW, SNNPR*

Key informants, including HEW supervisors and senior officials at health centers unanimously agreed that HEW salaries were inadequate. HEWs cater to a large population, often in topographically difficult terrains and on foot, so physical strain due to their job came up as a common theme and a constraint to their motivation. *"I still remember how horrible it was…the 4 hour walk in the mountains. It rains over us, and the sun burns so bad"*, remembers a leaver from SNNPR.

Material Factors

Material factors were also seen as important in influencing HEW motivation and retention. These often took the form of adequate drugs, equipment and infrastructure available at the health post. Such factors were found to be critical not only to support their daily work, but important to sustain the rapport and faith the community had in them by managing to do the

For peer review only - http://bmjopen.bmj.com/site/about/guidelines.xhtml

tasks entrusted to them. Sometimes facilities were perceived so lacking that faith was the only answer:

"Sometimes I support the labouring mothers by praying to Gebriel (Angel), because what we learn is different from what we apply. The materials that we have are inadequate; we only have delivery kit, which contains scissor, and cord tie. When a mother delivers at hospital, many things are provided to her and her baby, but here we have nothing to give her".

-Leaver, Tigray

In addition, HEWs and leavers suggested that material incentives such as motorcycles for transportation should be provided as part of their work package, to decrease their physical burden.

Furthermore, the gender of HEWs results in a double burden, as many mothers with infants mentioned that it was hard for them to do their daily tasks as a HEW, alongside caring for their infants.

"It is very difficult having a child. I leave from my home early morning at 6 am […] I may stay up to 6 pm, sometimes I don't even have time to drink water after coming back from field work . So, imagine doing all things having a baby"

-Leaver, Tigray

For HEWs with young children, the absence of childcare was a disincentive to continue in their jobs after giving birth.

HEWs also mentioned not always feeling safe in travelling to rural areas. *"Facilities like motor for transportation should be fulfilled. This security issue also needs attention since in rural areas females can be abused,"* stated a leaver from Tigray.

For peer review only - http://bmjopen.bmj.com/site/about/guidelines.xhtml

Non-material factors

Most importantly, HEWs and leavers mentioned highly appreciating the non-material factors such as appreciation from their communities and supervisors. The opportunity to improve community health, especially that of mothers and children, and gain their community's trust, respect and acceptance, was unanimously described as the top factor motivating them in their jobs.

"When I get the acceptance of healthy mothers and children, I am satisfied. Otherwise, the salary is not enough; the high workload is as I told you before".

- Active HEW, SNNPR

Sometimes, HEWs were not as easily accepted by their community, which demotivated them. Often respondents claimed that these demand-side barriers existed because of low levels of education and awareness among community members, which also led them to reject healthcare interventions such as family planning and latrine construction.

"The community's behaviour is difficult. For example, when we go to their home to educate them about environmental hygiene, they may close their door and leave from home. They say, oh! She is coming! When I enter through the front door, they will leave the house from the back door. It is for them but they do not understand. To teach them about something we will take many days. They have a shortage of knowledge."

- Leaver, Tigray

Other non-material demotivating factors were things that HEWs and leavers identified as lacking in HEW jobs. For example, the placement of HEWs in health posts, often far away from their hometowns where their husband and children are based, limited their motivation and retention. All three study populations agreed that the absence of opportunities to transfer to a facility closer to their family was frustrating, unfair, and led HEWs to leave their positions. *"This was my main reason to leave my job… Imagine that you can't meet with*

For peer review only - http://bmjopen.bmj.com/site/about/guidelines.xhtml

your husband as well as your children for a long time because there is no transfer (opportunity)", mentioned a leaver from Tigray.

In addition, two key issues around career progression were identified. First, HEWs that were keen to upskill to the next level[1] had to take a competitive exam in English – a language they are not generally proficient in and do not normally use in their jobs, and on topics in which they had not received enough training. The success rate for these exams was thus found to be low. HEWs complained that while many of them are excellent field workers with many years of experience in delivering healthcare, their inability to do well in an exam should not be the sole determinant of career progression.

The second key issue was for HEWs who did manage to upgrade to the next level, but despite upskilling, were expected to return to their old jobs at the health post. Many HEWs agreed that while after upgrading, their remuneration increased (or was expected to increase in the following months); they were expected to undertake the same tasks, in the same health post as before.

"After we get back from our level 4 study, we will be placed to the same kebele (village) as before. We need to be refreshed, be in a new place! Alongside with transfer, we should also be assigned to health centres (promoted to a higher health facility)",

-Active HEW, SNNPR

Another reason why HEWs and leavers felt de-motivated was the lack of support, oversight, and acknowledgement from supervisors and managers, who said that supervision was based on a model of faultfinding, not mentorship.

[1] The majority of HEWs are currently hired as level 3 (now increasingly level 4) health workers and according to HEP, HEWs have the opportunity to upskill to the next level after taking a competitive exam. After this, based on opportunities available and skills needed in the district, HEWs can further upgrade to diploma level courses in subjects like midwifery, and even complete a master's program in public health from government universities.

For peer review only - http://bmjopen.bmj.com/site/about/guidelines.xhtml

"…[…]. I was so tired that night that I could not clean all the blood and every mess (after single- headedly doing a delivery at the health post). Next morning the woreda (district) officials showed up and insulted me without considering what I have been through. It was so painful not to be understood to this level."

- *Leaver, SNNPR*

Intrinsic motivational factors

Many HEWs mentioned that the key reason for joining the profession was to serve the community where they were raised.

"Most of the time in our environment, the mothers don't use contraceptives, they don't give birth in health centres and they don't get ante-natal care. The mothers normally give birth in their home with a traditional birth attendant. Because of this, many mothers die. When I saw these types of problems in my community, I decided to become a HEW. "

- *Active HEW, SNNPR*

Some HEWs also insisted that financial incentives were less important than intrinsic factors and that the profession requires women to be truly dedicated to the community's health improvement, to survive in their jobs.

Many leavers mentioned having left their jobs out of frustration with challenging conditions but confessed to have really enjoyed working towards improving community health. *"Regarding the profession, health extension work itself has no concerns. I believe as a HEW you get to serve or work for the community which is great... it's the working conditions that are problematic,"* said a leaver from Tigray.

DISCUSSION

Our study findings from two regions in Ethiopia contribute new empirical evidence to the global knowledge and debate on factors influencing the motivation and retention of CHWs in LMICs. It is the first study to include the perspectives of those who had left their posts. Since we wanted to capture the individual experiences and behaviour of all three respondent

For peer review only - http://bmjopen.bmj.com/site/about/guidelines.xhtml

groups, we refrained from conducting focus group discussions and stuck to using in-depth interviews. Moreover, we believe that reasons why people leave their jobs or things they find unsatisfying are of sensitive nature, unlikely to be disclosed in a group of peers.

Many of the extrinsic motivational factors we identified, such as wages and allowances, were similar to those identified for CHWs in other settings (11, 24, 27, 33, 37, 38). For example, a study in Bangladesh reported lack of time to attend to their own children and other household responsibilities, insufficient profit/salary, and their families' disapproval as reasons cited by CHWs for leaving their posts (39). In Nigeria, village health workers reported low work satisfaction due to the lack of career advancement opportunities, low salaries and poor supervision (40).

Our study offers a number of new perspectives that we believe are valuable in Ethiopia and in other LMICs. Discussion around an adequate career path for CHWs in LMICs is ongoing (8, 33, 37). Despite WHO's repeated recommendations on a set career ladder for CHWs to be established in individual country contexts (8), the uptake has been low by governments. For example, in Ethiopia, there is evidence that the majority of HEWs are keen to take on more responsibility and upgrade to become nurses, pharmacy technicians and health administrators (41) but no such career path is offered to them. Access to and provision of upgrading and promotion opportunities was identified as one of top five measures to motivate HEWs and improve HEP services in another study (42). The need for and the value of career progression amongst CHWs to improve job satisfaction, was also a key topic of discussion at an international symposium on CHWs in 2019 (43). Moreover, the rate of attrition was reported to be nearly three times greater for higher level professionals like doctors and nurses, in comparison to other allied health professionals lower in the hierarchy. Factors identified as contributing towards these attrition rates were higher budget constraints, but also the lack of educational opportunities and poor career development (23).

For peer review only - http://bmjopen.bmj.com/site/about/guidelines.xhtml

Other material and non-material incentives affecting retention in this context were better living and working conditions that included their ability to live close to their family, have easy access to water and electricity at home and at work. According to a study published in 2007 (28), the living and working conditions of HEWs during early stages of HEP had not met basic standards. A more recent study suggested that many health posts were still missing basic infrastructure like water supply, electricity in 2012 (44). The mean availability of tracer items for basic facilities, infection prevention, malaria diagnosis, and essential medicines at health posts was 37, 29, 52 and 47%, respectively, according to data from a service availability and readiness assessment, in 2016. (45, 46)

Additionally, there is growing recognition of the importance of gender inclusiveness and equity in healthcare, which entails transforming the systems within which women work, such as highlighted in a recent report from the World Health Organization’s Gender Equity Hub (47). In a Cochrane review, socio-cultural norms that restrict movement of female CHWs and govern acceptable male-female communications were also identified as barriers to doing their jobs successfully (48). Jackson et al. (49) apply a gender lens to HEP and to the role of HEWs and conclude that by changing gender norms and reducing constraints to gender equality, HEP could have more transformative outcomes not just for HEWs but for the communities they serve. Since the majority of HEWs in Ethiopia are women of reproductive age, providing them with childcare, particularly for when they are away for house visits, could be a step forward in gender transforming their work environment.

Social behaviour and preferences of HEWs

While our evidence supports the importance of material incentives, we also identified other influences on social preferences of CHWs, which could help understand how they prioritize across multiple factors. Such insights could inform the development of new interventions to motivate and satisfy CHWs and retain them in the long term.

For peer review only - http://bmjopen.bmj.com/site/about/guidelines.xhtml

In this context, while conventional models have identified motivation as intrinsic and extrinsic, our empirical results identified two further additions – pro-social preferences as a non-material motivator, and social identity as a factor that could influence how CHWs trade among attributes. The social identity approach demonstrates how processes within an individual that influence behaviour are dependent upon interpersonal relationships and group memberships, as well as their perceived value and significance to the individual (50, 51). This approach states that when a person identifies as a member of a group, and when a given group identity is relevant to an individual; their behaviour becomes more focused towards what is seen to be in the group's interest, rather than their own (51, 52).

Thus, when workers define themselves in terms of a personal identity it could be expected that individual motivators such as personal advancement and financial incentives may be more influential. However, when defining themselves in terms of a social identity, motivators that impact on the group one identifies with, such as their status, standing and acceptance in the group may become more influential (53), like in the case of HEWs. This is a hypothesis that merits further empirical investigation. While the social identity approach is increasingly being applied in high-income countries (11, 53), it is less common in LMICs. To our knowledge, the inSCALE project, which operated in Uganda and Mozambique (11), is the first to use the social identity approach in a LMIC context to address these constraints in motivation of CHWs. Our study drew on formative research results from the inSCALE study and applied the social identity approach for establishing links between identification and motivation (54, 55) in the context of CHWs in Ethiopia.

It has already been recognised that non-material interventions to support CHWs can contribute substantially to creating a more satisfied health workforce that is able and willing to continue delivering quality healthcare to communities.(31, 49, 56, 57). In the Ethiopian context, focus particularly could be accorded to improve not just the availability of strategic resources such as mentoring and supervision, but the quality of support offered by often male supervisors to these female workers. Addressing HEW aspirations to progress in their

For peer review only - http://bmjopen.bmj.com/site/about/guidelines.xhtml

jobs by providing sufficient upgrading opportunities, tailored to their preferences and abilities, has good potential for improving their job satisfaction, reducing attrition. Clearly, positive community attitude towards HEWs is a key demand-side requirement for HEWs to stay motivated. We believe a good rapport between HEWs and the community often results when HEWs are capable of providing healthcare to the standards expected by the community. This of course is a function of having health posts with adequate infrastructure as well as well-trained HEWs. Equally important is that HEWs are emotionally satisfied, not having to live apart from their families due to the lack of transfer opportunities.

Future research should explore the development of interventions that can create and maintain trust between CHWs and the community. It could further be evaluated if a bottom-up approach that is designed with the inputs of CHWs and the community, is better tailored to the needs and realities of both.(25) In addition to health outcomes, policymakers should also invest in studying outcome measures such as competencies and self-esteem of health workers as this can have direct effects on their retention and indirect effects on the sustainable delivery of population health.

For peer review only

CONCLUSION

Our study showed that CHW jobs in LMICs including Ethiopia continue to be challenging, and incentives that align with their preferences have the potential to improve their motivation, influencing retention. However, modifying material incentives alone might not improve retention in the long term. Using empirical data from our study and theories of CHW motivation from the literature, we have demonstrated that CHWs identify themselves as members of a group (in this case their community and team). Thus, appealing to their social needs may represent a relatively more acceptable, potentially cost-effective and complementary strategy to the traditional approach of using financial incentive packages for improving retention, particularly in the long run in resource-constrained settings. These non-

For peer review only - http://bmjopen.bmj.com/site/about/guidelines.xhtml

material factors are important to be considered by government policymakers in resource constrained settings like Ethiopia that are struggling with critical health workforce shortages and inadequate health budgets. The voices of health workers can offer insights that may otherwise be missed and should thus be included while designing programs to improve retention.

DECLARATIONS

Ethics approval

Ethical approval was obtained from the London School of Hygiene and Tropical Medicine, UK (ref no. 16177), aswell as Addis Ababa University, Ethiopia (ref. no. 015/19/SPH) in March 2019.

Patient consent for publication

Not required

Data sharing statement

The data sets generated and analysed in the study are available on reasonable request made to the corresponding author.

Competing interests

None declared.

Funding

The Fieldwork for this study was funded by the Wellcome Trust (grant 212771/Z/18/Z), and the IDEAS project, London School of Hygiene and Tropical Medicine.

For peer review only

Author's contributions

All authors were involved in the original design of the qualitative study in Ethiopia. AW, FT, YH, YM conducted and translated all the interviews. DK, AE provided extensive in-country support. NA was the principal investigator who oversaw the fieldwork and conducted majority of the analysis, reviewed and approved by MQ and KH. NS provided expert guidance on manuscript development and analysis. All authors read and approved the final manuscript.

Acknowledgements

The authors would like to thank all the respondents who participated in the interviews.

REFERENCES

1. World Health Organization. Leave no one behind: Strengthening health systems for UHC and the SDGs in Africa. 2017.
2. Bhutta ZA, Lassi ZS, Pariyo G, Huicho L. Global experience of community health workers for delivery of health related millennium development goals: a systematic review, country case studies, and recommendations for integration into national health systems. Global health workforce Alliance. 2010;1(249):61.
3. Lewin S, Dick J, Pond P, Zwarenstein M, Aja GN, van Wyk BE, et al. Lay health workers in primary and community health care for maternal and child health and the management of infectious diseases Cochrane Database Syst Rev. 2005(1).
4. Perry H, Zulliger R. An overview of current evidence with recommendations for strengthening community health worker programs to accelerate progress in achieving the health-related Millennium Development Goals. . Baltimore: Johns Hopkins Bloomberg School of Public Health. 2012:84.
5. Perry HB, Zulliger R, Rogers MM. Community health workers in low-, middle-, and high-income countries: an overview of their history, recent evolution, and current effectiveness. Annual Review of Public Health 2014;35:399-421.
6. Cometto G, Ford N, Pfaffman-Zambruni J, Akl EA, Lehmann U, McPake B, et al. Health policy and system support to optimise community health worker programmes: an abridged WHO guideline. The Lancet Global Health. 2018;6(12):e1397-e404.
7. Lehmann U, Sanders D. Community health workers: what do we know about them. Bulletin of the World Health Organization 2007:1-42.
8. World Health Organization. WHO guideline on health policy and system support to optimize community health worker programmes. 2018.
9. Madeleine Ballard MHB, Jodi-Ann Burey, Jennifer Foth, Kevin Fiori Jr, Isaac Holeman, Ari Johnson, Serah Malaba, Daniel Palazuelos, Mallika Raghavan, Ash Rogers et al. .

For peer review only

For peer review only - http://bmjopen.bmj.com/site/about/guidelines.xhtml

Community Health Worker Assessment and Improvement Matrix (CHW AIM): Updated Program Functionality Matrix for Optimizing Community Health Programs. 2018.

10. Brown C, Lilford R, Griffiths F, Oppong-Darko P, Ndambo M, Okoh-Owusu M, et al. Case study of a method of development of a selection process for community health workers in sub-Saharan Africa. Human Resources for Health. 2019;17(1):75.

11. Strachan DL, Källander K, Nakirunda M, Ndima S, Muiambo A, Hill Z. Using theory and formative research to design interventions to improve community health worker motivation, retention and performance in Mozambique and Uganda. Human resources for health. 2015;13(1):25.

12. Bhattacharyya K, Winch P, LeBan K, Tien M. Community health worker incentives and disincentives: how they affect motivation retention and sustainability. 2001.

13. Mueller D, Kurowski C, Mills A. Managing health workforce performance. Component–literature review: determinants and levers of health worker motivation and satisfaction. Health Economics Financing Program London: London School of Hygiene Tropical Medicine International Health. 2005.

14. Chandler CI, Chonya S, Mtei F, Reyburn H, Whitty CJ. Motivation, money and respect: a mixed-method study of Tanzanian non-physician clinicians. Social science and medicine 2009;68(11):2078-88.

15. Agarwal S, Anaba U, Abuya T, Kintu R, Casseus A, Hossain S, et al. Understanding incentive preferences of community health workers using discrete choice experiments: a multicountry protocol for Kenya, Uganda, Bangladesh and Haiti. BMJ Open. 2019;9(12):e033601.

16. Shiratori S, Agyekum EO, Shibanuma A, Oduro A, Okawa S, Enuameh Y, et al. Motivation and incentive preferences of community health officers in Ghana: an economic behavioral experiment approach. Human resources for health. 2016;14(1):53.

17. Abdel-All M, Angell B, Jan S, Howell M, Howard K, Abimbola S, et al. What do community health workers want? Findings of a discrete choice experiment among Accredited Social Health Activists (ASHAs) in India. BMJ Glob Health. 2019;4(3):e001509-e.

18. Federal Ministry of Health (FMOH). Health Sector Strategic Plan (HSDP-III) 2005/6-2009/10. 2005 2 December 2019.

19. The World Bank. The Health Extension Program in Ethiopia 2012.

20. Federal Ministry of Health E. Health Sector Transformation Plan (HSTP) 2015/16 - 2019/20. 2015.

21. Wang H, Tesfaye R, NV Ramana G, Chekagn CT. Ethiopia health extension program: an institutionalized community approach for universal health coverage: The World Bank; 2016.

22. MERQ Consultancy Plc. National assessment of the ethiopian health extension program, abridged report 2019.

23. Hailemichael Y, Jira C, Girma B, Tushune K. Health workforce deployment, attrition and density in East wollega zone, Western ethiopia. Ethiop. 2010;20(1):15-23.

24. Kok MC, Kea AZ, Datiko DG, Broerse JE, Dieleman M, Taegtmeyer M, et al. A qualitative assessment of health extension workers' relationships with the community and health sector in Ethiopia: opportunities for enhancing maternal health performance. Hum Resour Health. 2015;13:80.

25. Kok MC, Ormel H, Broerse JEW, Kane S, Namakhoma I, Otiso L, et al. Optimising the benefits of community health workers' unique position between communities and the

For peer review only

For peer review only - http://bmjopen.bmj.com/site/about/guidelines.xhtml

health sector: A comparative analysis of factors shaping relationships in four countries. Glob Public Health. 2017;12(11):1404-32.

26. Kok MC, Kane SS, Tulloch O, Ormel H, Theobald S, Dieleman M, et al. How does context influence performance of community health workers in low- and middle-income countries? Evidence from the literature. Health Res Policy Syst. 2015;13:13.

27. Mohammed S, Tilahun M, Kote M, Mama M, Tamiru D. Validation of Health Extension Workers Job Motivation Scale in Gamo-Gofa Zone, Southern Ethiopia: A Cross-Sectional Study. Int Sch Res Notices. 2015;2015:250610.

28. Kitaw Y, Ye-Ebiyo Y, Said A, Desta H, Teklehaimanot A. Assessment of the training of the first intake of health extension workers. The Ethiopian Journal of Health Development. 2007;21(3).

29. Kane S, Kok M, Ormel H, Otiso L, Sidat M, Namakhoma I, et al. Limits and opportunities to community health worker empowerment: A multi-country comparative study. Social Science & Medicine. 2016;164:27-34.

30. Maes KC, Kohrt BA, Closser S. Culture, status and context in community health worker pay: pitfalls and opportunities for policy research. A commentary on Glenton et al. (2010). Social Science & Medicine. 2010;71(8):1375-8; discussion 9-80.

31. Maes K, Closser S, Kalofonos I. Listening to community health workers: how ethnographic research can inform positive relationships among community health workers, health institutions, and communities. Am J Public Health. 2014;104(5):e5-9.

32. Maes K, Closser S, Tesfaye Y, Gilbert Y, Abesha R. Volunteers in Ethiopia's women's development army are more deprived and distressed than their neighbors: cross-sectional survey data from rural Ethiopia. BMC Public Health. 2018;18(1):258.

33. Ormel H, Kok M, Kane S, Ahmed R, Chikaphupha K, Rashid SF, et al. Salaried and voluntary community health workers: exploring how incentives and expectation gaps influence motivation. Human Resources for Health. 2019;17(1):59.

34. Braun V, Clarke V. Using thematic analysis in psychology. Qualitative Research in Psychology. 2006;3(2):77-101.

35. QSR International Pty Ltd. Version 12. NVivo qualitative data analysis software2018.

36. Takemura T, Kielmann K, Blaauw D. Job preferences among clinical officers in public sector facilities in rural Kenya: a discrete choice experiment. Journal of Human resources for health. 2016;14(1):1.

37. Li L, Hu H, Zhou H, He C, Fan L, Liu X, et al. Work stress, work motivation and their effects on job satisfaction in community health workers: a cross-sectional survey in China. BMJ Open. 2014;4(6):e004897.

38. Chevalier C, Lapo A, O'Brien J, Wierzba TF. Why do village health workers drop out? World Health Forum. 1993;14(3):258-61.

39. Khan SH, Chowdhury A, Karim F, Barua MKJTHcs. Training and retaining Shasthyo Shebika: reasons for turnover of community health workers in Bangladesh. 1998;17(1):37-47.

40. Gray HH, Ciroma J. Reducing attrition among village health workers in rural Nigeria. Socio-Economic Planning Sciences. 1988;22(1):39-43.

41. Awash Teklehaimanot YK, Samuel Girma, Aklilu Seyoum, Yemane Ye-Ebiyo. Study of the working conditions of Health Extension Woekrs in Ethiopia. Ethiopian Journal of Health Development. 2007.

For peer review only

42. Centre for National Health Development in Ethiopia and Columbia University. Ethiopia Health Extension Program Evaluation Study, 2007-2010. Health post and HEWs performance survey. 2011.
43. International Centre for Diarrhoeal Disease Reserach B. CHW 2019 Symposium [Available from: http://chwsymposium2019.icddrb.org/.
44. Medhanyie A, Spigt M, Dinant G, Blanco R. Knowledge and performance of the Ethiopian health extension workers on antenatal and delivery care: a cross-sectional study. Human resources for health. 2012;10(1):44.
45. Ethiopian Public Health Institute (EPHI). Ethiopian Service Availability and Readiness Assessment 2016 Summary Report Addis Ababa, Ethiopia.
46. Assefa Y, Gelaw YA, Hill PS, Taye BW, Van Damme W. Community health extension program of Ethiopia, 2003–2018: successes and challenges toward universal coverage for primary healthcare services. Globalization and Health. 2019;15(1):24.
47. World Health Organization. Delivered by women, led by men: a gender and equity analysis of the global health and social workforce. 2019.
48. Sarin E, Lunsford SS. How female community health workers navigate work challenges and why there are still gaps in their performance: a look at female community health workers in maternal and child health in two Indian districts through a reciprocal determinism framework. Hum Resour Health. 2017;15(1):44.
49. Jackson R, Kilsby D, Hailemariam A. Gender exploitative and gender transformative aspects of employing Health Extension Workers under Ethiopia's Health Extension Program. Tropical Medicine & International Health. 2019;24(3):304-19.
50. Turner J, Reynolds K. Rediscovering social identity: core sources. The Story of Social Identity. 2010:13-32.
51. Lewis T. Assessing social identity and collective efficacy as theories of group motivation at work. The international journal of human resource management. 2011;22(04):963-80.
52. Postmes T, Haslam SA, Jans L. A single-item measure of social identification: Reliability, validity, and utility. British journal of social psychology. 2013;52(4):597-617.
53. Haslam SA. Psychology in organizations: Sage; 2004.
54. Van Dick R, Wagner U. Social identification among school teachers: Dimensions, foci, and correlates. European Journal of Work Organizational Psychology. 2002;11(2):129-49.
55. Wegge J, Van Dick R, Fisher GK, Wecking C, Moltzen K. Work motivation, organisational identification, and well-being in call centre work. Journal of Work Stress. 2006;20(1):60-83.
56. Jackson R, Hailemariam A. The Role of Health Extension Workers in Linking Pregnant Women With Health Facilities for Delivery in Rural and Pastoralist Areas of Ethiopia. Ethiop. 2016;26(5):471-8.
57. Saran I, Winn L, Kipkoech Kirui J, Menya D, Prudhomme O'Meara W. The relative importance of material and non-material incentives for community health workers: Evidence from a discrete choice experiment in Western Kenya. Social Science & Medicine. 2020;246:112726.

Figure 1: A framework of relationships between motivational factors, motivation and CHW work behaviour. Modified from Ormel et al

For peer review only - http://bmjopen.bmj.com/site/about/guidelines.xhtml

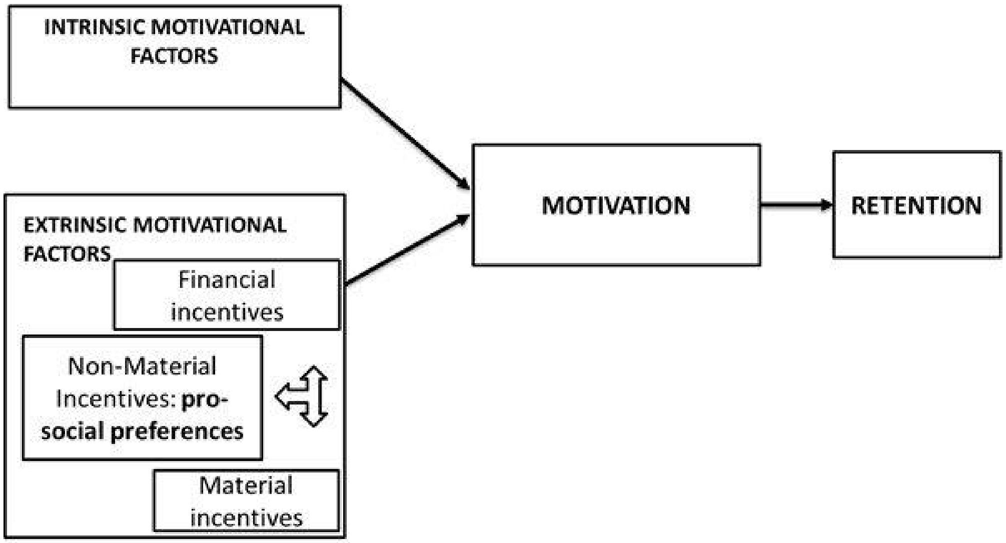

A framework of relationships between motivational factors, motivation and CHW work behaviour. Modified from Ormel et al

38x20mm (400 x 400 DPI)

Standards for Reporting Qualitative Research (SRQR)*

http://www.equator-network.org/reporting-guidelines/srqr/

Title and abstract

	Page/line no(s).
**Title** - Concise description of the nature and topic of the study Identifying the study as qualitative or indicating the approach (e.g., ethnography, grounded theory) or data collection methods (e.g., interview, focus group) is recommended	1/1-3
**Abstract** - Summary of key elements of the study using the abstract format of the intended publication; typically includes background, purpose, methods, results, and conclusions	2-3/28-60

Introduction

**Problem formulation** - Description and significance of the problem/phenomenon studied; review of relevant theory and empirical work; problem statement	4-5/81-134
**Purpose or research question** - Purpose of the study and specific objectives or questions	6/135-149

Methods

**Qualitative approach and research paradigm** - Qualitative approach (e.g., ethnography, grounded theory, case study, phenomenology, narrative research) and guiding theory if appropriate; identifying the research paradigm (e.g., postpositivist, constructivist/ interpretivist) is also recommended; rationale**	6/151-159
**Researcher characteristics and reflexivity** - Researchers' characteristics that may influence the research, including personal attributes, qualifications/experience, relationship with participants, assumptions, and/or presuppositions; potential or actual interaction between researchers' characteristics and the research questions, approach, methods, results, and/or transferability	8, 9/190-212
**Context** - Setting/site and salient contextual factors; rationale**	7/163-171
**Sampling strategy** - How and why research participants, documents, or events were selected; criteria for deciding when no further sampling was necessary (e.g., sampling saturation); rationale**	7/table 1 and lines 172-175
**Ethical issues pertaining to human subjects** - Documentation of approval by an appropriate ethics review board and participant consent, or explanation for lack thereof; other confidentiality and data security issues	Page 21/ 520-522 and page 8,9/190-207
**Data collection methods** - Types of data collected; details of data collection procedures including (as appropriate) start and stop dates of data collection and analysis, iterative process, triangulation of sources/methods, and modification of procedures in response to evolving study findings; rationale**	Page 6 line 151, page 8 lines 196-212, page 9 215-220-211

For peer review only - http://bmjopen.bmj.com/site/about/guidelines.xhtml

**Data collection instruments and technologies** - Description of instruments (e.g., interview guides, questionnaires) and devices (e.g., audio recorders) used for data collection; if/how the instrument(s) changed over the course of the study	Page 8/194-197
**Units of study** - Number and relevant characteristics of participants, documents, or events included in the study; level of participation (could be reported in results)	Page 7, 8/173-187
**Data processing** - Methods for processing data prior to and during analysis, including transcription, data entry, data management and security, verification of data integrity, data coding, and anonymization/de-identification of excerpts	Page 9 /207-212
**Data analysis** - Process by which inferences, themes, etc., were identified and developed, including the researchers involved in data analysis; usually references a specific paradigm or approach; rationale**	Page 9/ 215-220
**Techniques to enhance trustworthiness** - Techniques to enhance trustworthiness and credibility of data analysis (e.g., member checking, audit trail, triangulation); rationale**	Page 9/ 216-220

Results/findings

**Synthesis and interpretation** - Main findings (e.g., interpretations, inferences, and themes); might include development of a theory or model, or integration with prior research or theory	Pages 9-16/225-399
**Links to empirical data** - Evidence (e.g., quotes, field notes, text excerpts, photographs) to substantiate analytic findings	Pages 9-16/225-399

Discussion

**Integration with prior work, implications, transferability, and contribution(s) to the field** - Short summary of main findings; explanation of how findings and conclusions connect to, support, elaborate on, or challenge conclusions of earlier scholarship; discussion of scope of application/generalizability; identification of unique contribution(s) to scholarship in a discipline or field	Page 16 -20/ 401-502
**Limitations** - Trustworthiness and limitations of findings	Page 3/ 61-71

Other

**Conflicts of interest** - Potential sources of influence or perceived influence on study conduct and conclusions; how these were managed	Page21/532
**Funding** - Sources of funding and other support; role of funders in data collection, interpretation, and reporting	Page 21/535-536

*The authors created the SRQR by searching the literature to identify guidelines, reporting standards, and critical appraisal criteria for qualitative research; reviewing the reference lists of retrieved sources; and contacting experts to gain feedback. The SRQR aims to improve the transparency of all aspects of qualitative research by providing clear standards for reporting qualitative research.

For peer review only - http://bmjopen.bmj.com/site/about/guidelines.xhtml

**The rationale should briefly discuss the justification for choosing that theory, approach, method, or technique rather than other options available, the assumptions and limitations implicit in those choices, and how those choices influence study conclusions and transferability. As appropriate, the rationale for several items might be discussed together.

Reference:
O'Brien BC, Harris IB, Beckman TJ, Reed DA, Cook DA. **Standards for reporting qualitative research: a synthesis of recommendations.** *Academic Medicine*, Vol. 89, No. 9 / Sept 2014 DOI: 10.1097/ACM.0000000000000388

For peer review only

For peer review only - http://bmjopen.bmj.com/site/about/guidelines.xhtml
